# Supplementary material for: Deciphering European Sea Bass (Dicentrarchus labrax) Resistance to Nervous Necrosis Virus by Transcriptome Analysis from Early Infection Towards Establishment of Virus Carrier State
Source: Int J Mol Sci. 2025 Sep 21;26(18):9220. doi: 10.3390/ijms26189220 (PMC12470603; doi:10.3390/ijms26189220)
Supplement: Supplementary file 1 [file ijms-26-09220-s001.zip › Revised_Toubanaki et al._IJMS_manuscript-SI.pdf]

International Journal of
*Molecular Sciences*

MDPI

*Article*

# Deciphering European sea bass (Dicentrarchus Labrax) resistance to nervous necrosis virus by transcriptome analysis from early infection towards establishment of virus carrier state

**Dimitra K. Toubanaki [1], Odysseas-Panagiotis Tzortzatos [1], Antonia Efstathiou [1], Vasileios Bakopoulos [2] and Evdokia Karagouni [1,*]**

[1] Immunology of Infection Group, Department of Microbiology, Hellenic Pasteur Institute, 11521 Athens, Greece; dtouban@pasteur.gr (D.K.T.); ptzortzatos@pasteur.gr (O.-P.T.); toniaef@pasteur.gr (A.E.)
[2] Department of Marine Sciences, School of The Environment, University of the Aegean, University Hill, Lesvos, 81100 Mytilene, Greece; v.bakopoulos@aegean.gr (V.B.)
* Correspondence: ekaragouni@pasteur.gr (E.K.); Tel.: +30-210-647-8826 (E.K.)

## Supplementary material

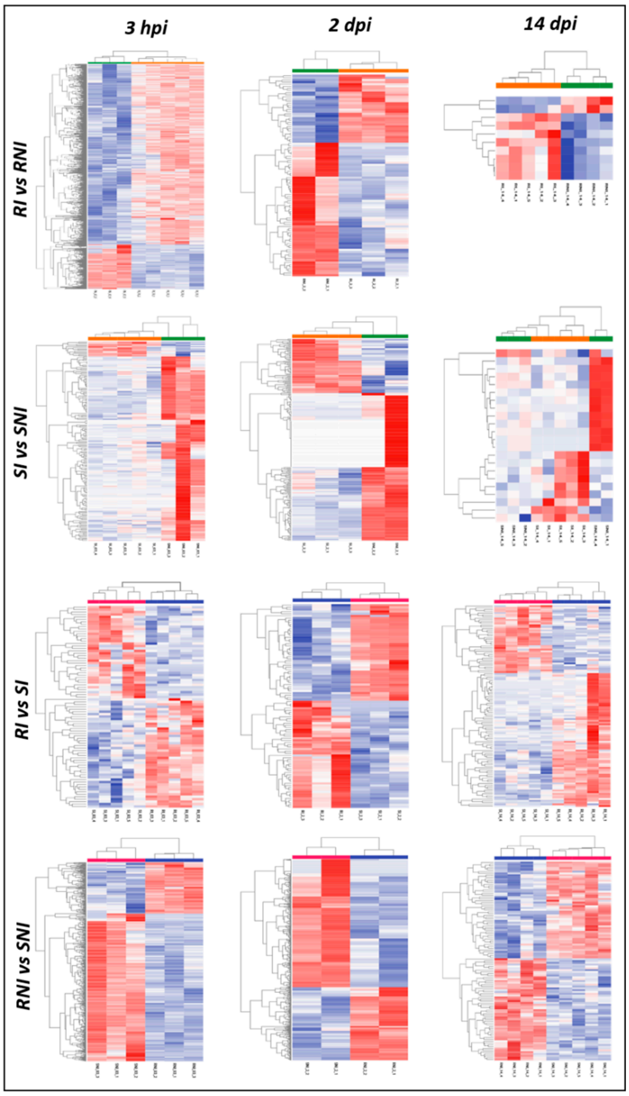


 https://doi.org/10.3390/ijms26189220

**Figure S1.** Cluster analysis of DEGs. Each column in the graph represents a sample, each row represents a gene with $log_2FC>2$, and the expression of genes in different samples is represented by different colors, with redder colors indicating higher expression and bluer colors indicating lower expression.

**Table S1.** Summary of the analyzed RNA-seq libraries total reads and alignment statistics.

| Sample | Total reads | Aligned one time | Aligned multiple times | Overall Alignment | Not Aligned |
|---|---|---|---|---|---|
| | | | | | |
| RI 3hpi_1 | 16,725,229 | 3,302,745 (19.8%) | 9,354,460 (55.9%) | 12,657,205 (75.7%) | 4,068,024 (24.3%) |
| RI 3hpi_2 | 15,792,647 | 3,485,273 (22.1%) | 8,101,891 (51.3%) | 11,587,164 (73.4%) | 4,205,483 (26.6%) |
| RI 3hpi_3 | 19,369,061 | 1,362,497 (7%) | 16,888,841 (87.2%) | 18,251,338 (94.2%) | 1,117,723 (5.8%) |
| RI 3hpi_4 | 14,574,502 | 1,248,665 (8.6%) | 12,001,881 (82.3%) | 13,250,546 (90.9%) | 1,323,956 (9.1%) |
| RI 3hpi_5 | 15,137,911 | 1,198,388 (7.9%) | 12,882,148 (85.1%) | 14,080,536 (93%) | 1,057,375 (7%) |
| RNI 3hpi_1 | 11,054,801 | 2,651,115 (24%) | 5,553,086 (50.2%) | 8,204,201 (74.2%) | 2,850,600 (25.8%) |
| RNI 3hpi_2 | 12,021,783 | 3,122,515 (26%) | 5,580,238 (46.4%) | 8,702,753 (72.4%) | 3,319,030 (27.6%) |
| RNI 3hpi_3 | 9,102,403 | 2,132,433 (23.4%) | 4,644,063 (51%) | 6,776,496 (74.5%) | 2,325,907 (25.5%) |
| SI 3hpi_1 | 8,376,527 | 2,778,929 (33.2%) | 2,984,934 (35.6%) | 5,763,863 (68.8%) | 2,612,664 (31.2%) |
| SI 3hpi_2 | 16,374,939 | 1,470,301 (9%) | 13,791,084 (84.2%) | 15,261,385 (93.2%) | 1,113,554 (6.8%) |
| SI 3hpi_3 | 17,463,217 | 1,505,108 (8.6%) | 15,565,325 (89.1%) | 17,070,433 (97.8%) | 392,784 (2.2%) |
| SI 3hpi_4 | 24,633,914 | 2,058,936 (8.4%) | 21,831,506 (88.6%) | 23,890,442 (97%) | 743,472 (3%) |
| SI 3hpi_5 | 15,968,236 | 2,067,728 (12.9%) | 12,815,291 (80.2%) | 14,883,019 (93.2%) | 1,085,217 (6.8%) |
| SNI 3hpi_1 | 11,838,302 | 2,488,653 (21%) | 5,599,661 (47.3%) | 8,088,314 (68.3%) | 3,749,988 (31.7%) |
| SNI 3hpi_2 | 7,480,560 | 1,613,719 (21.6%) | 3,361,581 (44.9%) | 4,975,300 (66.5%) | 2,505,260 (33.5%) |
| SNI 3hpi_3 | 6,653,708 | 1,651,931 (24.8%) | 2,783,610 (41.8%) | 4,435,541 (66.6%) | 2,218,167 (33.3%) |
| RI 2dpi_1 | 9,199,799 | 1,893,460 (20.6%) | 5,123,199 (55.7%) | 7,016,659 (76.3%) | 2,183,140 (23.7%) |
| RI 2dpi_2 | 18,139,621 | 1,422,561 (7.8%) | 15,346,522 (84.6%) | 16,769,083 (92.4%) | 1,370,538 (7.5%) |
| RI 2dpi_3 | 19,261,571 | 1,666,779 (8.6%) | 16,744,451 (86.9%) | 18,411,230 (95.6%) | 850,341 (4.4%) |
| RNI 2dpi_1 | 14,447,934 | 3,330,115 (23.5%) | 7,147,429 (49.5%) | 10,477,544 (72.5%) | 3,970,390 (27.5%) |
| RNI 2dpi_2 | 9,129,937 | 2,080,747 (22.8%) | 4,300,404 (47.1%) | 6,381,151 (69.9%) | 2,748,786 (30.1%) |
| SI 2dpi_1 | 16,138,181 | 1,254,832 (7.8%) | 13,693,202 (84.8%) | 14,948,034 (92.6%) | 1,190,147 (7.4%) |
| SI 2dpi_2 | 17,261,106 | 1,744,522 (10.1%) | 13,443,321 (77.9%) | 15,187,843 (88%) | 2,073,263 (12%) |
| SI 2dpi_3 | 22,378,376 | 1,451,120 (6.5%) | 19,480,175 (87.5%) | 20,931,295 (93.5%) | 1,447,081 (6.5%) |
| SNI 2dpi_1 | 10,924,705 | 1,940,230 (17.8%) | 5,616,665 (51.4%) | 7,556,895 (69.2%) | 3,367,810 (30.8%) |
| SNI 2dpi_2 | 11,206,870 | 2,187,498 (19.5%) | 5,651,058 (50.4%) | 7,838,556 (69.9%) | 3,368,314 (30.1%) |
| RI 14dpi_1 | 24,860,558 | 2,484,930 (10%) | 20,272,133 (81.5%) | 22,757,063 (91.5%) | 2,103,495 (8.5%) |
| RI 14dpi_2 | 23,822,187 | 2,316,145 (9.7%) | 20,120,746 (84.5%) | 22,436,891 (94.2%) | 1,385,296 (5.8%) |
| RI 14dpi_3 | 28,617,650 | 3,800,614 (13.3%) | 21,870,771 (76.4%) | 25,671,385 (89.7%) | 2,946,265 (10.3%) |
| RI 14dpi_4 | 14,400,548 | 1,281,626 (8.9%) | 11,537,276 (80.1%) | 12,818,902 (89%) | 1,581,646 (11%) |
| RI 14dpi_5 | 15,439,357 | 1,326,335(8.6%) | 12,999,082 (84.2%) | 14,325,417 (92.8%) | 1,113,940 (7.2%) |
| RNI 14dpi_1 | 13,151,000 | 1,410,213 (10.8%) | 10,668,956 (81.1%) | 12,079,169 (91.8%) | 1,071,831 (8.1%) |
| RNI 14dpi_2 | 17,669,423 | 2,011,744 (11.4%) | 14,378,261 (81.4%) | 16,390,005 (92.8%) | 1,279,418 (7.2%) |
| RNI 14dpi_3 | 15,990,321 | 1,556,658 (9.7%) | 13,460,641 (84.2%) | 15,017,299 (93.9%) | 973,022 (6.1%) |
| RNI 14dpi_4 | 15,007,899 | 1,477,793 (9.8%) | 13,022,981 (86.8%) | 14,500,774 (96.6%) | 507,125 (3.4%) |
| SI 14dpi_1 | 16,361,729 | 1,338,821 (8.2%) | 13,482,000 (82.4%) | 14,820,821 (90.6%) | 1,540,908 (9.4%) |
| SI 14dpi_2 | 15,416,229 | 1,571,489 (10.2%) | 11,996,325 (77.8%) | 13,567,814 (88%) | 1,848,415 (12%) |
| SI 14dpi_3 | 29,454,355 | 2,409,915 (8.2%) | 24,101,898 (81.8%) | 26,511,813 (90%) | 2,942,542 (10%) |
| SI 14dpi_4 | 14,388,534 | 1,332,154 (9.3%) | 11,773,030 (81,.8%) | 13,105,184 (91.1%) | 1,283,350 (8.9%) |
| SI 14dpi_5 | 17,088,312 | 1,765,369 (10.3%) | 12,886,678 (75.4%) | 14,652,047 (85.7%) | 2,436,265 (14.3%) |
| SNI 14dpi_1 | 11,080,406 | 1,275,852 (11.5%) | 9,047,271 (81.6%) | 10,323,123 (93.2%) | 757,283 (6.8%) |

| | | | | | |
|---|---|---|---|---|---|
| SNI 14dpi_2 | 21,093,407 | 2,061,023 (9.8%) | 17,626,734 (83.6%) | 19,687,757 (93.3%) | 1,405,650 (6.7%) |
| SNI 14dpi_3 | 20,452,275 | 1,988,475 (9.7%) | 16,745,998 (81.9%) | 18,734,473 (91.6%) | 1,717,802 (8.4%) |
| SNI 14dpi_4 | 16,901,162 | 2,289,598 (13.5%) | 13,960,522 (82.6%) | 16,250,120 (96.1%) | 651,042 (3.8%) |
| SNI 14dpi_5 | 20,915,008 | 1,901,941 (9.1%) | 17,889,843 (85.5%) | 19,791,784 (94.6%) | 1,123,224 (5.4%) |

**Table S2.** The Gene Ontology (GO) enriched terms of up-regulated DEGs in RI vs. RNI groups at 3 hpi, 2 and 14 dpi. BP: Biological process; MF: molecular function; CC: cellular component; FDR: False discovery rate; RI: resistant infected fish; RNI: resistant non-infected fish; hpi: hours post infection; dpi: days post infection.

| GO Term | GO Name | GO Category | p-value | FDR |
|---|---|---|---|---|
| | | **RI vs RNI 3 hpi** | | |
| GO:0002376 | immune system process | BP | 0,00E+00 | 0,00E+00 |
| GO:0023052 | signaling | BP | 0,00E+00 | 0,00E+00 |
| GO:0005886 | plasma membrane | CC | 2,01E-284 | 6,83E-282 |
| GO:0048856 | anatomical structure devel-opment | BP | 4,63E-264 | 1,57E-261 |
| GO:0005829 | cytosol | CC | 2,33E-234 | 7,91E-232 |
| GO:0030154 | cell differentiation | BP | 2,14E-199 | 7,28E-197 |
| GO:0016740 | transferase activity | MF | 4,95E-155 | 1,68E-152 |
| GO:0005615 | extracellular space | CC | 6,06E-149 | 2,06E-146 |
| GO:0140096 | catalytic activity, acting on a protein | MF | 5,64E-144 | 1,92E-141 |
| GO:0005654 | nucleoplasm | CC | 1,60E-142 | 5,45E-140 |
| GO:0006355 | regulation of DNA-tem-plated transcription | BP | 3,99E-129 | 1,36E-126 |
| GO:0016192 | vesicle-mediated transport | BP | 7,35E-115 | 2,50E-112 |
| GO:0003677 | DNA binding | MF | 2,11E-111 | 7,17E-109 |
| GO:0012501 | programmed cell death | BP | 1,34E-108 | 4,57E-106 |
| GO:0007010 | cytoskeleton organization | BP | 1,20E-97 | 4,06E-95 |
| GO:0065003 | protein-containing complex assembly | BP | 1,31E-94 | 4,44E-92 |
| GO:0005739 | mitochondrion | CC | 2,15E-93 | 7,32E-91 |
| GO:0055085 | transmembrane transport | BP | 3,63E-87 | 1,23E-84 |
| GO:0007155 | cell adhesion | BP | 1,43E-84 | 4,86E-82 |
| GO:0048870 | cell motility | BP | 6,64E-79 | 2,26E-76 |
| GO:0098542 | defense response to other or-ganism | BP | 3,09E-77 | 1,05E-74 |
| GO:0140110 | transcription regulator activ-ity | MF | 1,12E-75 | 3,82E-73 |
| GO:0005783 | endoplasmic reticulum | CC | 4,88E-73 | 1,66E-70 |
| GO:0006886 | intracellular protein transport | BP | 1,15E-72 | 3,91E-70 |
| GO:0005794 | Golgi apparatus | CC | 3,82E-70 | 1,30E-67 |
| GO:0005215 | transporter activity | MF | 3,26E-66 | 1,11E-63 |
| GO:0061024 | membrane organization | BP | 2,45E-65 | 8,33E-63 |
| GO:0016491 | oxidoreductase activity | MF | 4,43E-57 | 1,50E-54 |
| GO:0060090 | molecular adaptor activity | MF | 1,18E-54 | 4,02E-52 |
| GO:0030163 | protein catabolic process | BP | 1,10E-53 | 3,75E-51 |
| GO:0060089 | molecular transducer activ-ity | MF | 1,52E-52 | 5,15E-50 |

| | | | | |
|---|---|---|---|---|
| GO:0008092 | cytoskeletal protein binding | MF | 1,84E-52 | 6,26E-50 |
| GO:0006629 | lipid metabolic process | BP | 1,02E-51 | 3,45E-49 |
| GO:0050877 | nervous system process | BP | 1,91E-51 | 6,51E-49 |
| GO:0140657 | ATP-dependent activity | MF | 2,11E-51 | 7,17E-49 |
| GO:0034330 | cell junction organization | BP | 2,98E-45 | 1,01E-42 |
| GO:0005768 | endosome | CC | 1,03E-44 | 3,49E-42 |
| GO:0006914 | autophagy | BP | 3,64E-40 | 1,24E-37 |
| GO:0055086 | nucleobase-containing small molecule metabolic process | BP | 1,83E-39 | 6,21E-37 |
| GO:0008270 | zinc ion binding | MF | 5,02E-39 | 1,71E-36 |
| GO:0006325 | chromatin organization | BP | 5,72E-39 | 1,94E-36 |
| GO:0007005 | mitochondrion organization | BP | 8,44E-39 | 2,87E-36 |
| GO:0003723 | RNA binding | MF | 1,38E-38 | 4,68E-36 |
| GO:0005815 | microtubule organizing center | CC | 3,31E-38 | 1,13E-35 |
| GO:0006091 | generation of precursor metabolites and energy | BP | 1,50E-37 | 5,10E-35 |
| GO:0140097 | catalytic activity, acting on DNA | MF | 1,10E-36 | 3,75E-34 |
| GO:0006954 | inflammatory response | BP | 1,44E-36 | 4,90E-34 |
| GO:0005975 | carbohydrate metabolic process | BP | 6,34E-35 | 2,16E-32 |
| GO:0005764 | lysosome | CC | 5,36E-34 | 1,82E-31 |
| GO:0003012 | muscle system process | BP | 1,06E-33 | 3,60E-31 |
| GO:0005730 | nucleolus | CC | 3,91E-33 | 1,33E-30 |
| GO:0006281 | DNA repair | BP | 1,68E-30 | 5,72E-28 |
| GO:0005198 | structural molecule activity | MF | 2,20E-30 | 7,49E-28 |
| GO:0016071 | mRNA metabolic process | BP | 7,64E-30 | 2,60E-27 |
| GO:0006310 | DNA recombination | BP | 8,78E-29 | 2,99E-26 |
| GO:0000228 | nuclear chromosome | CC | 2,12E-27 | 7,21E-25 |
| GO:0008289 | lipid binding | MF | 3,54E-27 | 1,20E-24 |
| GO:0030198 | extracellular matrix organization | BP | 6,96E-25 | 2,37E-22 |
| GO:0003924 | GTPase activity | MF | 8,24E-24 | 2,80E-21 |
| GO:0005635 | nuclear envelope | CC | 3,91E-22 | 1,33E-19 |
| GO:0072659 | protein localization to plasma membrane | BP | 5,56E-22 | 1,89E-19 |
| GO:0140098 | catalytic activity, acting on RNA | MF | 1,10E-21 | 3,72E-19 |
| GO:0006520 | amino acid metabolic process | BP | 3,14E-21 | 1,07E-18 |
| GO:0006790 | sulfur compound metabolic process | BP | 6,00E-20 | 2,04E-17 |
| GO:0006913 | nucleocytoplasmic transport | BP | 6,00E-20 | 2,04E-17 |
| GO:0005929 | cilium | CC | 2,21E-19 | 7,50E-17 |
| GO:0044782 | cilium organization | BP | 4,70E-19 | 1,60E-16 |
| GO:0007163 | establishment/maintenance of cell polarity | BP | 7,28E-19 | 2,48E-16 |
| GO:0042393 | histone binding | MF | 1,68E-18 | 5,71E-16 |
| GO:0000910 | cytokinesis | BP | 8,31E-18 | 2,83E-15 |
| GO:0006260 | DNA replication | BP | 9,94E-18 | 3,38E-15 |

| | | | | |
|---|---|---|---|---|
| GO:0048018 | receptor ligand activity | MF | 2,56E-17 | 8,70E-15 |
| GO:0007059 | chromosome segregation | BP | 3,35E-17 | 1,14E-14 |
| GO:0003013 | circulatory system process | BP | 1,13E-16 | 3,83E-14 |
| GO:0016874 | ligase activity | MF | 1,36E-16 | 4,63E-14 |
| GO:0042254 | ribosome biogenesis | BP | 8,74E-16 | 2,97E-13 |
| GO:0007018 | microtubule-based movement | BP | 1,54E-15 | 5,25E-13 |
| GO:0042060 | wound healing | BP | 8,49E-15 | 2,89E-12 |
| GO:0140014 | mitotic nuclear division | BP | 1,99E-14 | 6,75E-12 |
| GO:0005525 | GTP binding | MF | 2,62E-12 | 8,92E-10 |
| GO:0005777 | peroxisome | CC | 2,64E-12 | 8,97E-10 |
| GO:0016829 | lyase activity | MF | 2,64E-12 | 8,97E-10 |
| GO:0031012 | extracellular matrix | CC | 8,65E-12 | 2,94E-09 |
| GO:0140053 | mitochondrial gene expression | BP | 1,05E-11 | 3,57E-09 |
| GO:0140013 | meiotic nuclear division | BP | 1,16E-11 | 3,95E-09 |
| GO:0006486 | protein glycosylation | BP | 1,16E-11 | 3,95E-09 |
| GO:0003774 | cytoskeletal motor activity | MF | 3,72E-11 | 1,26E-08 |
| GO:0006399 | tRNA metabolic process | BP | 1,75E-10 | 5,93E-08 |
| GO:0007040 | lysosome organization | BP | 7,04E-09 | 2,39E-06 |
| GO:0031047 | regulatory ncRNA-mediated gene silencing | BP | 1,76E-08 | 5,99E-06 |
| GO:0007031 | peroxisome organization | BP | 4,82E-08 | 1,64E-05 |
| GO:0003014 | renal system process | BP | 2,96E-07 | 1,01E-04 |
| GO:0045182 | translation regulator activity | MF | 5,57E-07 | 1,89E-04 |
| GO:0009617 | response to bacterium | BP | 8,00E-07 | 2,72E-04 |
| GO:0022600 | digestive system process | BP | 1,75E-06 | 5,96E-04 |
| GO:0032200 | telomere organization | BP | 8,19E-06 | 2,78E-03 |
| GO:0140223 | general transcription initiation factor activity | MF | 1,33E-05 | 4,51E-03 |
| GO:0098754 | detoxification | BP | 1,33E-05 | 4,51E-03 |
| GO:0003016 | respiratory system process | BP | 1,52E-05 | 5,15E-03 |
| GO:0038024 | cargo receptor activity | MF | 1,52E-05 | 5,15E-03 |
| GO:0006575 | cellular modified amino acid metabolic process | BP | 1,71E-05 | 5,81E-03 |
| GO:0005840 | ribosome | CC | 3,84E-05 | 1,31E-02 |
| GO:0098631 | cell adhesion mediator activity | MF | 6,32E-05 | 2,15E-02 |
| GO:0006457 | protein folding | BP | 8,21E-05 | 2,79E-02 |
| | **RI vs RNI 2 dpi** | | | |
| GO:0005886 | plasma membrane | CC | 5,21E-20 | 1,27E-17 |
| GO:0002376 | immune system process | BP | 3,80E-18 | 9,24E-16 |
| GO:0048856 | anatomical structure development | BP | 1,24E-17 | 3,02E-15 |
| GO:0005615 | extracellular space | CC | 6,18E-17 | 1,50E-14 |
| GO:0005829 | cytosol | CC | 1,10E-14 | 2,67E-12 |
| GO:0016740 | transferase activity | MF | 9,71E-13 | 2,36E-10 |
| GO:0140657 | ATP-dependent activity | MF | 2,92E-11 | 7,08E-09 |
| GO:0003774 | cytoskeletal motor activity | MF | 2,92E-11 | 7,08E-09 |
| GO:0008092 | cytoskeletal protein binding | MF | 9,57E-10 | 2,33E-07 |

| | | | | |
|---|---|---|---|---|
| GO:0007010 | cytoskeleton organization | BP | 1,38E-09 | 3,36E-07 |
| GO:0005815 | microtubule organizing center | CC | 1,73E-09 | 4,21E-07 |
| GO:0030154 | cell differentiation | BP | 3,51E-09 | 8,52E-07 |
| GO:0023052 | signaling | BP | 5,89E-09 | 1,43E-06 |
| GO:0007059 | chromosome segregation | BP | 6,92E-09 | 1,68E-06 |
| GO:0140097 | catalytic activity, acting on DNA | MF | 1,73E-08 | 4,20E-06 |
| GO:0007018 | microtubule-based movement | BP | 3,46E-08 | 8,40E-06 |
| GO:0003012 | muscle system process | BP | 3,46E-08 | 8,40E-06 |
| GO:0048018 | receptor ligand activity | MF | 9,66E-08 | 2,35E-05 |
| GO:0012501 | programmed cell death | BP | 1,45E-07 | 3,52E-05 |
| GO:0006355 | regulation of DNA-templated transcription | BP | 2,07E-07 | 5,02E-05 |
| GO:0006260 | DNA replication | BP | 1,49E-06 | 3,62E-04 |
| GO:0043167 | ion binding | MF | 1,49E-06 | 3,62E-04 |
| GO:0005730 | nucleolus | CC | 4,47E-06 | 1,09E-03 |
| GO:0016491 | oxidoreductase activity | MF | 4,47E-06 | 1,09E-03 |
| GO:0005198 | structural molecule activity | MF | 8,92E-06 | 2,17E-03 |
| GO:0140014 | mitotic nuclear division | BP | 8,92E-06 | 2,17E-03 |
| GO:0005694 | chromosome | CC | 8,92E-06 | 2,17E-03 |
| GO:0006886 | intracellular protein transport | BP | 2,23E-05 | 5,41E-03 |
| GO:1901564 | organonitrogen compound metabolic process | BP | 2,23E-05 | 5,41E-03 |
| GO:0140096 | catalytic activity, acting on a protein | MF | 2,23E-05 | 5,41E-03 |
| GO:0005654 | nucleoplasm | CC | 4,15E-05 | 1,01E-02 |
| GO:0016192 | vesicle-mediated transport | BP | 5,33E-05 | 1,30E-02 |
| GO:0022414 | reproductive process | BP | 5,33E-05 | 1,30E-02 |
| GO:0097159 | organic cyclic compound binding | MF | 5,33E-05 | 1,30E-02 |
| GO:0016787 | hydrolase activity | MF | 6,66E-05 | 1,62E-02 |
| GO:0031410 | cytoplasmic vesicle | CC | 6,66E-05 | 1,62E-02 |
| GO:0048870 | cell motility | BP | 8,14E-05 | 1,98E-02 |
| **RI vs RNI 14 dpi** | | | | |
| GO:0002376 | immune system process | BP | 5,88E-19 | 5,11E-17 |
| GO:0005615 | extracellular space | CC | 3,79E-15 | 3,30E-13 |
| GO:0098542 | defense response to other organism | BP | 1,96E-11 | 1,70E-09 |
| GO:0140110 | transcription regulator activity | MF | 3,10E-04 | 2,70E-02 |
| GO:0060090 | molecular adaptor activity | MF | 3,10E-04 | 2,70E-02 |
| GO:0006091 | generation of precursor metabolites and energy | BP | 3,10E-04 | 2,70E-02 |
| GO:0000228 | nuclear chromosome | CC | 3,10E-04 | 2,70E-02 |
| GO:0023052 | signaling | BP | 3,10E-04 | 2,70E-02 |
| GO:0048856 | anatomical structure development | BP | 3,10E-04 | 2,70E-02 |

| | | | | |
|---|---|---|---|---|
| GO:0005654 | nucleoplasm | CC | 3,10E-04 | 2,70E-02 |
| GO:0016071 | mRNA metabolic process | BP | 3,10E-04 | 2,70E-02 |
| GO:0140098 | catalytic activity, acting on RNA | MF | 3,10E-04 | 2,70E-02 |
| GO:0055086 | nucleobase-containing small molecule metabolic process | BP | 3,10E-04 | 2,70E-02 |
| GO:0022414 | reproductive process | BP | 3,10E-04 | 2,70E-02 |
| GO:0006355 | regulation of DNA-templated transcription | BP | 3,10E-04 | 2,70E-02 |
| GO:1901135 | carbohydrate derivative metabolic process | BP | 3,10E-04 | 2,70E-02 |
| GO:0003677 | DNA binding | MF | 3,10E-04 | 2,70E-02 |
| GO:0005739 | mitochondrion | CC | 3,10E-04 | 2,70E-02 |
| GO:0030154 | cell differentiation | BP | 3,10E-04 | 2,70E-02 |
| GO:0016787 | hydrolase activity | MF | 3,10E-04 | 2,70E-02 |

**Table S3.** The Gene Ontology (GO) enriched terms of down-regulated DEGs in RI vs. RNI groups at 3 hpi, 2 and 14 dpi. BP: Biological process; MF: molecular function; CC: cellular component; FDR: False discovery rate; RI: resistant infected fish; RNI: resistant non-infected fish; hpi: hours post infection; dpi: days post infection.

| GO Term | GO Name | GO Category | p-value | FDR |
|---|---|---|---|---|
| | | **RI vs RNI 3 hpi** | | |
| GO:0048856 | anatomical structure development | BP | 2,27E-144 | 7,73E-142 |
| GO:0030154 | cell differentiation | BP | 3,39E-111 | 1,15E-108 |
| GO:0023052 | signaling | BP | 5,52E-94 | 1,88E-91 |
| GO:0006355 | regulation of DNA-templated transcription | BP | 1,18E-88 | 4,03E-86 |
| GO:0005886 | plasma membrane | CC | 5,28E-73 | 1,80E-70 |
| GO:0005829 | cytosol | CC | 5,39E-69 | 1,83E-66 |
| GO:0022414 | reproductive process | BP | 2,80E-61 | 9,53E-59 |
| GO:0005654 | nucleoplasm | CC | 9,59E-59 | 3,26E-56 |
| GO:0002376 | immune system process | BP | 4,91E-45 | 1,67E-42 |
| GO:0140110 | transcription regulator activity | MF | 1,97E-44 | 6,70E-42 |
| GO:0048870 | cell motility | BP | 5,98E-44 | 2,03E-41 |
| GO:0003723 | RNA binding | MF | 4,55E-43 | 1,55E-40 |
| GO:0003677 | DNA binding | MF | 4,64E-41 | 1,58E-38 |
| GO:0012501 | programmed cell death | BP | 3,83E-40 | 1,30E-37 |
| GO:0005615 | extracellular space | CC | 2,40E-37 | 8,15E-35 |
| GO:0065003 | protein-containing complex assembly | BP | 1,41E-35 | 4,80E-33 |
| GO:0050877 | nervous system process | BP | 4,46E-35 | 1,52E-32 |
| GO:0005783 | endoplasmic reticulum | CC | 1,78E-34 | 6,06E-32 |
| GO:0016192 | vesicle-mediated transport | BP | 1,87E-33 | 6,35E-31 |
| GO:0016740 | transferase activity | MF | 2,74E-32 | 9,31E-30 |
| GO:0006629 | lipid metabolic process | BP | 4,39E-31 | 1,49E-28 |
| GO:0007155 | cell adhesion | BP | 2,21E-29 | 7,50E-27 |
| GO:0005794 | Golgi apparatus | CC | 1,39E-28 | 4,72E-26 |
| GO:0016071 | mRNA metabolic process | BP | 2,94E-28 | 9,99E-26 |
| GO:0140096 | catalytic activity, acting on a protein | MF | 1,69E-26 | 5,74E-24 |

| | | | | |
|---|---|---|---|---|
| GO:0060089 | molecular transducer activity | MF | 7,95E-26 | 2,70E-23 |
| GO:0042060 | wound healing | BP | 7,44E-25 | 2,53E-22 |
| GO:0007010 | cytoskeleton organization | BP | 1,39E-24 | 4,73E-22 |
| GO:0006325 | chromatin organization | BP | 6,01E-24 | 2,04E-21 |
| GO:0060090 | molecular adaptor activity | MF | 4,69E-23 | 1,59E-20 |
| GO:0003013 | circulatory system process | BP | 4,65E-20 | 1,58E-17 |
| GO:0055085 | transmembrane transport | BP | 7,97E-20 | 2,71E-17 |
| GO:0006954 | inflammatory response | BP | 3,67E-19 | 1,25E-16 |
| GO:0034330 | cell junction organization | BP | 4,76E-18 | 1,62E-15 |
| GO:0031012 | extracellular matrix | CC | 4,85E-17 | 1,65E-14 |
| GO:0006886 | intracellular protein transport | BP | 1,49E-16 | 5,05E-14 |
| GO:0098542 | defense response to other organism | BP | 3,42E-16 | 1,16E-13 |
| GO:0008092 | cytoskeletal protein binding | MF | 4,43E-16 | 1,50E-13 |
| GO:0005739 | mitochondrion | CC | 4,71E-15 | 1,60E-12 |
| GO:1901135 | carbohydrate derivative metabolic process | BP | 1,26E-14 | 4,27E-12 |
| GO:0005768 | endosome | CC | 1,63E-14 | 5,56E-12 |
| GO:0008289 | lipid binding | MF | 3,87E-14 | 1,32E-11 |
| GO:0045182 | translation regulator activity | MF | 4,20E-14 | 1,43E-11 |
| GO:0030163 | protein catabolic process | BP | 1,14E-13 | 3,87E-11 |
| GO:0016491 | oxidoreductase activity | MF | 2,20E-13 | 7,48E-11 |
| GO:0005215 | transporter activity | MF | 1,11E-12 | 3,76E-10 |
| GO:0005730 | nucleolus | CC | 3,56E-12 | 1,21E-09 |
| GO:0005198 | structural molecule activity | MF | 2,80E-11 | 9,53E-09 |
| GO:0003012 | muscle system process | BP | 6,25E-11 | 2,12E-08 |
| GO:0031047 | regulatory ncRNA-mediated gene silencing | BP | 9,14E-11 | 3,11E-08 |
| GO:0061024 | membrane organization | BP | 1,23E-10 | 4,17E-08 |
| GO:0048018 | receptor ligand activity | MF | 1,88E-10 | 6,39E-08 |
| GO:0030198 | extracellular matrix organization | BP | 1,75E-09 | 5,95E-07 |
| GO:0007005 | mitochondrion organization | BP | 2,50E-09 | 8,51E-07 |
| GO:0002181 | cytoplasmic translation | BP | 4,38E-09 | 1,49E-06 |
| GO:0007163 | establishment or maintenance of cell polarity | BP | 5,69E-09 | 1,94E-06 |
| GO:0006790 | sulfur compound metabolic process | BP | 7,76E-09 | 2,64E-06 |
| GO:0006913 | nucleocytoplasmic transport | BP | 7,76E-09 | 2,64E-06 |
| GO:0005635 | nuclear envelope | CC | 1,39E-08 | 4,72E-06 |
| GO:0005975 | carbohydrate metabolic process | BP | 1,56E-08 | 5,30E-06 |
| GO:0055086 | nucleobase-containing small molecule metabolic process | BP | 3,50E-08 | 1,19E-05 |
| GO:0007059 | chromosome segregation | BP | 6,16E-08 | 2,09E-05 |
| GO:0005929 | cilium | CC | 1,11E-07 | 3,77E-05 |
| GO:0005525 | GTP binding | MF | 2,52E-07 | 8,56E-05 |
| GO:0003924 | GTPase activity | MF | 3,12E-07 | 1,06E-04 |
| GO:0006575 | cellular modified amino acid metabolic process | BP | 3,17E-07 | 1,08E-04 |
| GO:0006914 | autophagy | BP | 4,49E-07 | 1,53E-04 |

| | | | | |
|---|---|---|---|---|
| GO:0022600 | digestive system process | BP | 5,37E-07 | 1,83E-04 |
| GO:0007018 | microtubule-based movement | BP | 6,67E-07 | 2,27E-04 |
| GO:0006260 | DNA replication | BP | 1,17E-06 | 3,99E-04 |
| GO:0016209 | antioxidant activity | MF | 1,68E-06 | 5,72E-04 |
| GO:0050886 | endocrine process | BP | 1,68E-06 | 5,72E-04 |
| GO:0005764 | lysosome | CC | 1,97E-06 | 6,69E-04 |
| GO:0006457 | protein folding | BP | 5,91E-06 | 2,01E-03 |
| GO:0032200 | telomere organization | BP | 9,74E-06 | 3,31E-03 |
| GO:0140657 | ATP-dependent activity | MF | 1,77E-05 | 6,00E-03 |
| GO:0005815 | microtubule organizing center | CC | 3,64E-05 | 1,24E-02 |
| GO:0140098 | catalytic activity, acting on RNA | MF | 3,75E-05 | 1,28E-02 |
| GO:0000228 | nuclear chromosome | CC | 9,11E-05 | 3,10E-02 |
| GO:0006766 | vitamin metabolic process | BP | 1,12E-04 | 3,81E-02 |
| GO:0140014 | mitotic nuclear division | BP | 1,29E-04 | 4,39E-02 |
| | **RI vs RNI 2 dpi** | | | |
| GO:0048856 | anatomical structure development | BP | 3,03E-53 | 7,36E-51 |
| GO:0023052 | signaling | BP | 1,71E-34 | 4,16E-32 |
| GO:0030154 | cell differentiation | BP | 2,66E-29 | 6,45E-27 |
| GO:0048870 | cell motility | BP | 9,29E-23 | 2,26E-20 |
| GO:0007010 | cytoskeleton organization | BP | 1,20E-21 | 2,92E-19 |
| GO:0005829 | cytosol | CC | 8,39E-21 | 2,04E-18 |
| GO:0031410 | cytoplasmic vesicle | CC | 3,56E-20 | 8,66E-18 |
| GO:0005886 | plasma membrane | CC | 4,06E-20 | 9,86E-18 |
| GO:0008092 | cytoskeletal protein binding | MF | 3,91E-19 | 9,49E-17 |
| GO:0003677 | DNA binding | MF | 2,92E-18 | 7,11E-16 |
| GO:0016192 | vesicle-mediated transport | BP | 1,31E-17 | 3,19E-15 |
| GO:0022414 | reproductive process | BP | 1,31E-17 | 3,19E-15 |
| GO:0006355 | regulation of DNA-templated transcription | BP | 4,37E-17 | 1,06E-14 |
| GO:0065003 | protein-containing complex assembly | BP | 1,66E-16 | 4,03E-14 |
| GO:0005654 | nucleoplasm | CC | 4,62E-15 | 1,12E-12 |
| GO:0012501 | programmed cell death | BP | 1,38E-14 | 3,36E-12 |
| GO:0016740 | transferase activity | MF | 7,59E-14 | 1,84E-11 |
| GO:0002376 | immune system process | BP | 2,81E-13 | 6,82E-11 |
| GO:0005856 | cytoskeleton | CC | 2,81E-13 | 6,82E-11 |
| GO:0050877 | nervous system process | BP | 4,42E-13 | 1,07E-10 |
| GO:0048018 | receptor ligand activity | MF | 4,11E-12 | 9,98E-10 |
| GO:0006954 | inflammatory response | BP | 3,22E-11 | 7,84E-09 |
| GO:0140110 | transcription regulator activity | MF | 1,61E-10 | 3,91E-08 |
| GO:0005794 | Golgi apparatus | CC | 1,61E-10 | 3,91E-08 |
| GO:0006886 | intracellular protein transport | BP | 4,82E-10 | 1,17E-07 |
| GO:0140096 | catalytic activity, acting on a protein | MF | 4,82E-10 | 1,17E-07 |
| GO:0060089 | molecular transducer activity | MF | 1,39E-08 | 3,37E-06 |
| GO:1901135 | carbohydrate derivative metabolic process | BP | 5,54E-08 | 1,35E-05 |
| GO:0006629 | lipid metabolic process | BP | 5,54E-08 | 1,35E-05 |

| | | | | |
|---|---|---|---|---|
| GO:0060090 | molecular adaptor activity | MF | 5,54E-08 | 1,35E-05 |
| GO:0055085 | transmembrane transport | BP | 5,54E-08 | 1,35E-05 |
| GO:0005929 | cilium | CC | 5,54E-08 | 1,35E-05 |
| GO:0007018 | microtubule-based movement | BP | 2,76E-07 | 6,70E-05 |
| GO:0003012 | muscle system process | BP | 2,76E-07 | 6,70E-05 |
| GO:0005635 | nuclear envelope | CC | 5,87E-06 | 1,43E-03 |
| GO:0007005 | mitochondrion organization | BP | 5,87E-06 | 1,43E-03 |
| GO:0003924 | GTPase activity | MF | 5,87E-06 | 1,43E-03 |
| GO:0005783 | endoplasmic reticulum | CC | 5,87E-06 | 1,43E-03 |
| GO:0007155 | cell adhesion | BP | 1,76E-05 | 4,27E-03 |
| GO:0030163 | protein catabolic process | BP | 1,76E-05 | 4,27E-03 |
| GO:0006325 | chromatin organization | BP | 1,76E-05 | 4,27E-03 |
| GO:0044281 | small molecule metabolic process | BP | 1,76E-05 | 4,27E-03 |
| GO:0000228 | nuclear chromosome | CC | 1,76E-05 | 4,27E-03 |
| GO:0034330 | cell junction organization | BP | 1,76E-05 | 4,27E-03 |
| GO:0008289 | lipid binding | MF | 1,76E-05 | 4,27E-03 |
| GO:0005215 | transporter activity | MF | 1,76E-05 | 4,27E-03 |
| GO:0006310 | DNA recombination | BP | 1,76E-05 | 4,27E-03 |
| GO:0061024 | membrane organization | BP | 1,76E-05 | 4,27E-03 |
| GO:0005198 | structural molecule activity | MF | 3,51E-05 | 8,53E-03 |
| GO:0140014 | mitotic nuclear division | BP | 3,51E-05 | 8,53E-03 |
| GO:0140097 | catalytic activity, acting on DNA | MF | 5,84E-05 | 1,42E-02 |
| GO:0140657 | ATP-dependent activity | MF | 8,74E-05 | 2,12E-02 |
| GO:0003774 | cytoskeletal motor activity | MF | 8,74E-05 | 2,12E-02 |
| GO:0005615 | extracellular space | CC | 1,63E-04 | 3,95E-02 |
| **RI vs RNI 14 dpi** | | | | |
| GO:0016491 | oxidoreductase activity | MF | 7,75E-05 | 6,74E-03 |
| GO:0043226 | organelle | CC | 3,87E-04 | 3,37E-02 |

**Table S4.** The Gene Ontology (GO) enriched terms of up-regulated DEGs in SI vs. SNI groups at 3 hpi, 2 and 14 dpi. BP: Biological process; MF: molecular function; CC: cellular component; FDR: False discovery rate; SI: susceptible infected fish; SNI: susceptible non-infected fish; hpi: hours post infection; dpi: days post infection.

| GO Term | GO Name | GO Category | p-value | FDR |
|---|---|---|---|---|
| **SI vs SNI 3 hpi** | | | | |
| GO:0005886 | plasma membrane | CC | 2,26E-09 | 5,56E-07 |
| GO:0016787 | hydrolase activity | MF | 1,53E-07 | 3,76E-05 |
| GO:0002376 | immune system process | BP | 2,01E-07 | 4,94E-05 |
| GO:0005576 | extracellular region | CC | 7,68E-07 | 1,89E-04 |
| GO:0005829 | cytosol | CC | 1,13E-06 | 2,79E-04 |
| GO:0005730 | nucleolus | CC | 1,40E-06 | 3,46E-04 |
| GO:0005515 | protein binding | MF | 1,70E-06 | 4,19E-04 |
| GO:0044238 | primary metabolic process | BP | 2,44E-06 | 6,00E-04 |
| GO:0006790 | sulfur compound metabolic process | BP | 3,51E-06 | 8,64E-04 |
| GO:0048856 | anatomical structure development | BP | 4,91E-06 | 1,21E-03 |

| | | | | |
|---|---|---|---|---|
| GO:0005654 | nucleoplasm | CC | 1,54E-05 | 3,79E-03 |
| GO:0044085 | cellular component biogenesis | BP | 5,38E-05 | 1,32E-02 |
| GO:0097159 | organic cyclic compound binding | MF | 6,43E-05 | 1,58E-02 |
| GO:0006810 | transport | BP | 1,15E-04 | 2,83E-02 |
| GO:0043170 | macromolecule metabolic process | BP | 1,30E-04 | 3,20E-02 |
| | | **SI vs SNI 2 dpi** | | |
| GO:0005525 | GTP binding | MF | 1,40E-40 | 3,79E-38 |
| GO:0002376 | immune system process | BP | 2,62E-22 | 7,10E-20 |
| GO:0005829 | cytosol | CC | 3,95E-16 | 1,07E-13 |
| GO:1901135 | carbohydrate derivative metabolic process | BP | 6,98E-16 | 1,89E-13 |
| GO:0055086 | nucleobase-containing small molecule metabolic process | BP | 6,98E-16 | 1,89E-13 |
| GO:0098542 | defense response to other organism | BP | 1,94E-15 | 5,27E-13 |
| GO:0003924 | GTPase activity | MF | 3,81E-14 | 1,03E-11 |
| GO:0005615 | extracellular space | CC | 4,50E-13 | 1,22E-10 |
| GO:0005811 | lipid droplet | CC | 4,79E-13 | 1,30E-10 |
| GO:0023052 | signaling | BP | 6,28E-13 | 1,70E-10 |
| GO:0140657 | ATP-dependent activity | MF | 1,67E-12 | 4,53E-10 |
| GO:0048856 | anatomical structure development | BP | 1,73E-11 | 4,70E-09 |
| GO:0005886 | plasma membrane | CC | 1,20E-09 | 3,25E-07 |
| GO:0005783 | endoplasmic reticulum | CC | 1,58E-09 | 4,28E-07 |
| GO:0005739 | mitochondrion | CC | 3,38E-08 | 9,16E-06 |
| GO:0016740 | transferase activity | MF | 3,38E-08 | 9,16E-06 |
| GO:0016491 | oxidoreductase activity | MF | 6,12E-08 | 1,66E-05 |
| GO:0140096 | catalytic activity, acting on a protein | MF | 6,12E-08 | 1,66E-05 |
| GO:0005856 | cytoskeleton | CC | 1,03E-07 | 2,78E-05 |
| GO:0006629 | lipid metabolic process | BP | 1,30E-07 | 3,51E-05 |
| GO:0030154 | cell differentiation | BP | 1,03E-06 | 2,79E-04 |
| GO:0045182 | translation regulator activity | MF | 6,06E-06 | 1,64E-03 |
| GO:0016070 | RNA metabolic process | BP | 7,96E-06 | 2,16E-03 |
| GO:0012501 | programmed cell death | BP | 7,96E-06 | 2,16E-03 |
| GO:0140097 | catalytic activity, acting on DNA | MF | 1,81E-05 | 4,92E-03 |
| GO:0065003 | protein-containing complex assembly | BP | 2,17E-05 | 5,87E-03 |
| GO:0044782 | cilium organization | BP | 3,62E-05 | 9,81E-03 |
| GO:0003723 | RNA binding | MF | 3,62E-05 | 9,81E-03 |
| GO:0005929 | cilium | CC | 3,62E-05 | 9,81E-03 |
| GO:0006790 | sulfur compound metabolic process | BP | 9,02E-05 | 2,45E-02 |
| GO:0005654 | nucleoplasm | CC | 1,26E-04 | 3,42E-02 |
| GO:0005215 | transporter activity | MF | 1,68E-04 | 4,55E-02 |
| | | **SI vs SNI 14 dpi** | | |

| | | | | |
|---|---|---|---|---|
| GO:0061024 | membrane organization | BP | 1,02E-13 | 1,72E-11 |
| GO:0005634 | nucleus | CC | 1,03E-09 | 1,72E-07 |
| GO:0030154 | cell differentiation | BP | 1,03E-09 | 1,72E-07 |
| GO:0048856 | anatomical structure development | BP | 2,46E-09 | 4,14E-07 |
| GO:0023052 | signaling | BP | 3,52E-09 | 5,91E-07 |
| GO:0007005 | mitochondrion organization | BP | 1,08E-07 | 1,82E-05 |
| GO:0003924 | GTPase activity | MF | 1,08E-07 | 1,82E-05 |
| GO:0065003 | protein-containing complex assembly | BP | 3,24E-07 | 5,45E-05 |
| GO:0005739 | mitochondrion | CC | 3,24E-07 | 5,45E-05 |
| GO:0098542 | defense response to other organism | BP | 3,24E-07 | 5,45E-05 |
| GO:0008092 | cytoskeletal protein binding | MF | 1,62E-06 | 2,72E-04 |
| GO:0005856 | cytoskeleton | CC | 3,02E-06 | 5,08E-04 |

**Table S5.** The Gene Ontology (GO) enriched terms of down-regulated DEGs in SI vs. SNI groups at 3hpi, 2 and 14 dpi. BP: Biological process; MF: molecular function; CC: cellular component; FDR: False discovery rate; SI: susceptible infected fish; SNI: susceptible non-infected fish; hpi: hours post infection; dpi: days post infection.

| GO Term | GO Name | GO Category | p-value | FDR |
|---|---|---|---|---|
| | | **SI vs SNI 3 hpi** | | |
| GO:0048856 | anatomical structure development | BP | 4,25E-146 | 1,05E-143 |
| GO:0003012 | muscle system process | BP | 2,47E-91 | 6,08E-89 |
| GO:0030154 | cell differentiation | BP | 1,03E-90 | 2,53E-88 |
| GO:0005829 | cytosol | CC | 5,96E-85 | 1,47E-82 |
| GO:0023052 | signaling | BP | 1,78E-80 | 4,38E-78 |
| GO:0008092 | cytoskeletal protein binding | MF | 9,98E-80 | 2,45E-77 |
| GO:0005886 | plasma membrane | CC | 4,82E-68 | 1,19E-65 |
| GO:0007010 | cytoskeleton organization | BP | 2,46E-61 | 6,05E-59 |
| GO:0005615 | extracellular space | CC | 6,85E-60 | 1,69E-57 |
| GO:0005783 | endoplasmic reticulum | CC | 1,12E-47 | 2,75E-45 |
| GO:0016192 | vesicle-mediated transport | BP | 2,45E-46 | 6,02E-44 |
| GO:0003677 | DNA binding | MF | 2,06E-45 | 5,06E-43 |
| GO:0003013 | circulatory system process | BP | 2,06E-45 | 5,06E-43 |
| GO:0048870 | cell motility | BP | 2,06E-45 | 5,06E-43 |
| GO:0002376 | immune system process | BP | 2,23E-44 | 5,48E-42 |
| GO:0065003 | protein-containing complex assembly | BP | 4,30E-44 | 1,06E-41 |
| GO:0006355 | regulation of DNA-templated transcription | BP | 4,30E-44 | 1,06E-41 |
| GO:0012501 | programmed cell death | BP | 7,49E-42 | 1,84E-39 |
| GO:0050877 | nervous system process | BP | 6,84E-41 | 1,68E-38 |
| GO:0016740 | transferase activity | MF | 6,25E-33 | 1,54E-30 |
| GO:0005198 | structural molecule activity | MF | 6,94E-32 | 1,71E-29 |
| GO:0007155 | cell adhesion | BP | 6,94E-32 | 1,71E-29 |
| GO:0140096 | catalytic activity, acting on a protein | MF | 1,22E-29 | 2,99E-27 |

| | | | | |
|---|---|---|---|---|
| GO:1901135 | carbohydrate derivative metabolic process | BP | 1,69E-28 | 4,17E-26 |
| GO:0042060 | wound healing | BP | 2,12E-27 | 5,22E-25 |
| GO:0022414 | reproductive process | BP | 2,74E-26 | 6,75E-24 |
| GO:0055085 | transmembrane transport | BP | 2,74E-26 | 6,75E-24 |
| GO:0140110 | transcription regulator activity | MF | 3,67E-25 | 9,03E-23 |
| GO:0140657 | ATP-dependent activity | MF | 4,38E-24 | 1,08E-21 |
| GO:0055086 | nucleobase-containing small molecule metabolic process | BP | 6,91E-22 | 1,70E-19 |
| GO:0005654 | nucleoplasm | CC | 4,12E-21 | 1,01E-18 |
| GO:0006091 | generation of precursor metabolites and energy | BP | 1,08E-20 | 2,65E-18 |
| GO:0006629 | lipid metabolic process | BP | 1,08E-20 | 2,65E-18 |
| GO:0003774 | cytoskeletal motor activity | MF | 1,08E-20 | 2,65E-18 |
| GO:0006954 | inflammatory response | BP | 1,83E-18 | 4,50E-16 |
| GO:0005975 | carbohydrate metabolic process | BP | 1,83E-18 | 4,50E-16 |
| GO:0060089 | molecular transducer activity | MF | 1,83E-18 | 4,50E-16 |
| GO:0008289 | lipid binding | MF | 1,83E-18 | 4,50E-16 |
| GO:0005794 | Golgi apparatus | CC | 3,09E-16 | 7,59E-14 |
| GO:0098542 | defense response to other organism | BP | 3,09E-16 | 7,59E-14 |
| GO:0061024 | membrane organization | BP | 3,09E-16 | 7,59E-14 |
| GO:0005215 | transporter activity | MF | 2,46E-15 | 6,04E-13 |
| GO:0000278 | mitotic cell cycle | BP | 5,17E-14 | 1,27E-11 |
| GO:0005739 | mitochondrion | CC | 5,17E-14 | 1,27E-11 |
| GO:0051604 | protein maturation | BP | 5,17E-14 | 1,27E-11 |
| GO:0060090 | molecular adaptor activity | MF | 5,17E-14 | 1,27E-11 |
| GO:0005635 | nuclear envelope | CC | 8,61E-12 | 2,12E-09 |
| GO:0007005 | mitochondrion organization | BP | 8,61E-12 | 2,12E-09 |
| GO:0034330 | cell junction organization | BP | 8,61E-12 | 2,12E-09 |
| GO:0006310 | DNA recombination | BP | 8,61E-12 | 2,12E-09 |
| GO:0030198 | extracellular matrix organization | BP | 8,61E-12 | 2,12E-09 |
| GO:0048018 | receptor ligand activity | MF | 5,14E-11 | 1,26E-08 |
| GO:0030163 | protein catabolic process | BP | 5,14E-11 | 1,26E-08 |
| GO:0006886 | intracellular protein transport | BP | 1,42E-09 | 3,50E-07 |
| GO:0031012 | extracellular matrix | CC | 1,42E-09 | 3,50E-07 |
| GO:0016491 | oxidoreductase activity | MF | 1,42E-09 | 3,50E-07 |
| GO:0006575 | cellular modified amino acid metabolic process | BP | 1,42E-09 | 3,50E-07 |
| GO:0000910 | cytokinesis | BP | 1,42E-09 | 3,50E-07 |
| GO:0005815 | microtubule organizing center | CC | 1,42E-09 | 3,50E-07 |
| GO:0006520 | amino acid metabolic process | BP | 7,08E-09 | 1,74E-06 |
| GO:0140097 | catalytic activity, acting on DNA | MF | 7,08E-09 | 1,74E-06 |
| GO:0006790 | sulfur compound metabolic process | BP | 2,11E-08 | 5,20E-06 |
| GO:0007163 | establishment or maintenance of cell polarity | BP | 2,34E-07 | 5,76E-05 |
| GO:0005764 | lysosome | CC | 9,32E-07 | 2,29E-04 |
| GO:0019905 | syntaxin binding | MF | 3,82E-05 | 9,40E-03 |

| | | | | |
|---|---|---|---|---|
| GO:0046983 | protein dimerization activity | MF | 3,82E-05 | 9,40E-03 |
| GO:0003014 | renal system process | BP | 3,82E-05 | 9,40E-03 |
| GO:0003016 | respiratory system process | BP | 3,82E-05 | 9,40E-03 |
| GO:0005768 | endosome | CC | 3,82E-05 | 9,40E-03 |
| GO:0000228 | nuclear chromosome | CC | 3,82E-05 | 9,40E-03 |
| GO:0006913 | nucleocytoplasmic transport | BP | 3,82E-05 | 9,40E-03 |
| GO:0016853 | isomerase activity | MF | 3,82E-05 | 9,40E-03 |
| GO:0016829 | lyase activity | MF | 3,82E-05 | 9,40E-03 |
| GO:0005929 | cilium | CC | 3,82E-05 | 9,40E-03 |
| GO:0003924 | GTPase activity | MF | 1,14E-04 | 2,81E-02 |
| **SI vs SNI 2 dpi** | | | | |
| GO:0023052 | signaling | BP | 7,64E-90 | 2,07E-87 |
| GO:0005615 | extracellular space | CC | 2,06E-85 | 5,58E-83 |
| GO:0005886 | plasma membrane | CC | 3,57E-78 | 9,69E-76 |
| GO:0048856 | anatomical structure development | BP | 2,29E-77 | 6,19E-75 |
| GO:0002376 | immune system process | BP | 5,12E-59 | 1,39E-56 |
| GO:0005829 | cytosol | CC | 1,28E-58 | 3,46E-56 |
| GO:0030154 | cell differentiation | BP | 1,05E-55 | 2,85E-53 |
| GO:0005198 | structural molecule activity | MF | 1,38E-44 | 3,74E-42 |
| GO:0065003 | protein-containing complex assembly | BP | 3,45E-39 | 9,35E-37 |
| GO:0003677 | DNA binding | MF | 6,11E-38 | 1,66E-35 |
| GO:0016192 | vesicle-mediated transport | BP | 7,04E-38 | 1,91E-35 |
| GO:0098542 | defense response to other organism | BP | 4,84E-36 | 1,31E-33 |
| GO:0098772 | molecular function regulator activity | MF | 1,50E-33 | 4,06E-31 |
| GO:0042060 | wound healing | BP | 2,54E-32 | 6,89E-30 |
| GO:0048870 | cell motility | BP | 2,54E-32 | 6,89E-30 |
| GO:0005783 | endoplasmic reticulum | CC | 1,96E-31 | 5,31E-29 |
| GO:0007010 | cytoskeleton organization | BP | 2,56E-31 | 6,94E-29 |
| GO:0006629 | lipid metabolic process | BP | 6,05E-30 | 1,64E-27 |
| GO:0005856 | cytoskeleton | CC | 7,65E-28 | 2,07E-25 |
| GO:0006954 | inflammatory response | BP | 4,23E-27 | 1,15E-24 |
| GO:0051604 | protein maturation | BP | 6,45E-27 | 1,75E-24 |
| GO:0008092 | cytoskeletal protein binding | MF | 6,45E-27 | 1,75E-24 |
| GO:0012501 | programmed cell death | BP | 2,24E-26 | 6,08E-24 |
| GO:0016491 | oxidoreductase activity | MF | 1,15E-23 | 3,11E-21 |
| GO:0140096 | catalytic activity, acting on a protein | MF | 1,15E-23 | 3,11E-21 |
| GO:0061024 | membrane organization | BP | 1,21E-23 | 3,28E-21 |
| GO:0060089 | molecular transducer activity | MF | 7,82E-23 | 2,12E-20 |
| GO:0006355 | regulation of DNA-templated transcription | BP | 7,82E-23 | 2,12E-20 |
| GO:0005794 | Golgi apparatus | CC | 1,73E-21 | 4,70E-19 |
| GO:0007155 | cell adhesion | BP | 1,73E-21 | 4,70E-19 |
| GO:0006325 | chromatin organization | BP | 2,46E-20 | 6,68E-18 |
| GO:0005739 | mitochondrion | CC | 1,55E-19 | 4,20E-17 |
| GO:0016740 | transferase activity | MF | 1,55E-19 | 4,20E-17 |

| | | | | |
|---|---|---|---|---|
| GO:0050877 | nervous system process | BP | 2,45E-19 | 6,64E-17 |
| GO:0008289 | lipid binding | MF | 2,45E-19 | 6,64E-17 |
| GO:0022414 | reproductive process | BP | 1,34E-18 | 3,63E-16 |
| GO:0006886 | intracellular protein transport | BP | 3,81E-18 | 1,03E-15 |
| GO:0055085 | transmembrane transport | BP | 3,41E-17 | 9,23E-15 |
| GO:0006520 | amino acid metabolic process | BP | 5,85E-16 | 1,58E-13 |
| GO:0140110 | transcription regulator activity | MF | 8,93E-14 | 2,42E-11 |
| GO:0005694 | chromosome | CC | 8,93E-14 | 2,42E-11 |
| GO:0006310 | DNA recombination | BP | 8,93E-14 | 2,42E-11 |
| GO:0005975 | carbohydrate metabolic process | BP | 6,21E-13 | 1,68E-10 |
| GO:0030163 | protein catabolic process | BP | 6,21E-13 | 1,68E-10 |
| GO:0005215 | transporter activity | MF | 2,47E-12 | 6,70E-10 |
| GO:0031012 | extracellular matrix | CC | 1,35E-11 | 3,67E-09 |
| GO:0030198 | extracellular matrix organization | BP | 1,35E-11 | 3,67E-09 |
| GO:0005764 | lysosome | CC | 8,08E-11 | 2,19E-08 |
| GO:0006091 | generation of precursor metabolites and energy | BP | 8,08E-11 | 2,19E-08 |
| GO:1901135 | carbohydrate derivative metabolic process | BP | 1,47E-10 | 3,99E-08 |
| GO:0055086 | nucleobase-containing small molecule metabolic process | BP | 1,47E-10 | 3,99E-08 |
| GO:0005654 | nucleoplasm | CC | 2,81E-10 | 7,62E-08 |
| GO:0003013 | circulatory system process | BP | 2,04E-09 | 5,54E-07 |
| GO:0034330 | cell junction organization | BP | 1,02E-08 | 2,75E-06 |
| GO:0006790 | sulfur compound metabolic process | BP | 3,03E-08 | 8,22E-06 |
| GO:0000278 | mitotic cell cycle | BP | 3,07E-07 | 8,31E-05 |
| GO:0007005 | mitochondrion organization | BP | 3,07E-07 | 8,31E-05 |
| GO:0005777 | peroxisome | CC | 3,07E-07 | 8,31E-05 |
| GO:0003924 | GTPase activity | MF | 4,16E-07 | 1,13E-04 |
| GO:0005730 | nucleolus | CC | 1,22E-06 | 3,31E-04 |
| GO:0006281 | DNA repair | BP | 4,57E-05 | 1,24E-02 |
| GO:0006766 | vitamin metabolic process | BP | 4,57E-05 | 1,24E-02 |
| GO:0003014 | renal system process | BP | 4,57E-05 | 1,24E-02 |
| GO:0031386 | protein tag activity | MF | 4,57E-05 | 1,24E-02 |
| GO:0050886 | endocrine process | BP | 4,57E-05 | 1,24E-02 |
| GO:0007031 | peroxisome organization | BP | 4,57E-05 | 1,24E-02 |
| GO:0005768 | endosome | CC | 4,57E-05 | 1,24E-02 |
| GO:0007163 | establishment or maintenance of cell polarity | BP | 4,57E-05 | 1,24E-02 |
| GO:0060090 | molecular adaptor activity | MF | 4,57E-05 | 1,24E-02 |
| GO:0031047 | regulatory ncRNA-mediated gene silencing | BP | 4,57E-05 | 1,24E-02 |
| GO:0016829 | lyase activity | MF | 4,57E-05 | 1,24E-02 |
| GO:0098631 | cell adhesion mediator activity | MF | 4,57E-05 | 1,24E-02 |
| GO:0007018 | microtubule-based movement | BP | 1,37E-04 | 3,70E-02 |
| GO:0005840 | ribosome | CC | 1,37E-04 | 3,70E-02 |
| **SI vs SNI 14 dpi** | | | | |
| GO:0005886 | plasma membrane | CC | 9,09E-24 | 1,53E-21 |

| | | | | |
|---|---|---|---|---|
| GO:0023052 | signaling | BP | 1,36E-22 | 2,29E-20 |
| GO:0005856 | cytoskeleton | CC | 1,17E-19 | 1,97E-17 |
| GO:0048856 | anatomical structure development | BP | 3,51E-19 | 5,90E-17 |
| GO:0007010 | cytoskeleton organization | BP | 1,35E-17 | 2,27E-15 |
| GO:0005829 | cytosol | CC | 8,10E-17 | 1,36E-14 |
| GO:0003012 | muscle system process | BP | 3,87E-14 | 6,50E-12 |
| GO:0008092 | cytoskeletal protein binding | MF | 5,80E-13 | 9,75E-11 |
| GO:0005634 | nucleus | CC | 1,35E-12 | 2,27E-10 |
| GO:0030154 | cell differentiation | BP | 1,35E-12 | 2,27E-10 |
| GO:0055086 | nucleobase-containing small molecule metabolic process | BP | 9,99E-11 | 1,68E-08 |
| GO:1901135 | carbohydrate derivative metabolic process | BP | 9,99E-11 | 1,68E-08 |
| GO:0005975 | carbohydrate metabolic process | BP | 9,99E-11 | 1,68E-08 |
| GO:0006091 | generation of precursor metabolites and energy | BP | 9,99E-11 | 1,68E-08 |
| GO:0002376 | immune system process | BP | 3,99E-10 | 6,71E-08 |
| GO:0140096 | catalytic activity, acting on a protein | MF | 3,99E-10 | 6,71E-08 |
| GO:0050877 | nervous system process | BP | 3,99E-10 | 6,71E-08 |
| GO:0012501 | programmed cell death | BP | 3,99E-10 | 6,71E-08 |
| GO:0005198 | structural molecule activity | MF | 2,34E-07 | 3,94E-05 |
| GO:0005694 | chromosome | CC | 2,34E-07 | 3,94E-05 |
| GO:0005215 | transporter activity | MF | 2,34E-07 | 3,94E-05 |
| GO:0055085 | transmembrane transport | BP | 2,34E-07 | 3,94E-05 |
| GO:0005615 | extracellular space | CC | 2,34E-07 | 3,94E-05 |
| GO:0003013 | circulatory system process | BP | 2,34E-07 | 3,94E-05 |
| GO:0048870 | cell motility | BP | 2,34E-07 | 3,94E-05 |
| GO:0012505 | endomembrane system | CC | 2,34E-07 | 3,94E-05 |
| GO:0060089 | molecular transducer activity | MF | 7,03E-07 | 1,18E-04 |
| GO:0016740 | transferase activity | MF | 7,03E-07 | 1,18E-04 |
| GO:0006950 | response to stress | BP | 1,40E-06 | 2,36E-04 |
| GO:0061024 | membrane organization | BP | 3,51E-06 | 5,90E-04 |
| GO:0016787 | hydrolase activity | MF | 3,51E-06 | 5,90E-04 |

**Table S6.** Enriched pathway categories on KEGG databases for RI vs. RNI groups at 3 hpi, 2 and 14 dpi. RI: resistant infected fish; RNI: resistant non-infected fish; hpi: hours post infection; dpi: days post infection.

| Pathway top category | Pathway class | Enriched pathway | 3 hpi | 2 dpi | 14 dpi |
|---|---|---|---|---|---|
| **Human Diseases** | Infectious disease: viral | Influenza A | ✓ | ✓ | ✓ |
| | | Coronavirus disease - COVID-19 | ✓ | ✓ | ✓ |
| | | Herpes simplex virus 1 infection | ✓ | | |
| | | Hepatitis C | ✓ | ✓ | |
| | | Human immunodeficiency virus 1 infection | ✓ | | |
| | | Human cytomegalovirus infection | ✓ | | ✓ |
| | | Human T-cell leukemia virus 1 infection | ✓ | | |
| | | Measles | ✓ | ✓ | |

| | | | | | |
|---|---|---|---|---|---|
| | | Hepatitis B | ✓ | ✓ | |
| Human Diseases | Infectious disease: bacterial | Pertussis | ✓ | ✓ | ✓ |
| | | Yersinia infection | ✓ | ✓ | ✓ |
| | | Salmonella infection | ✓ | ✓ | ✓ |
| | | Pathogenic Escherichia coli infection | ✓ | ✓ | ✓ |
| | | Shigellosis | ✓ | ✓ | ✓ |
| | | Staphylococcus aureus infection | ✓ | | ✓ |
| | | Legionellosis | ✓ | | |
| | | Bacterial invasion of epithelial cells | ✓ | ✓ | |
| | | Tuberculosis | ✓ | ✓ | |
| | | Vibrio cholerae infection | ✓ | ✓ | |
| | | Epithelial cell signaling in Helicobacter pylori infection | ✓ | ✓ | |
| Human Diseases | Infectious disease: parasitic | Malaria | ✓ | | |
| | | Chagas disease | ✓ | ✓ | |
| | | African trypanosomiasis | | ✓ | |
| | | Leishmaniasis | | ✓ | |
| | | Toxoplasmosis | | ✓ | |
| Human Diseases | Immune disease | Allograft rejection | ✓ | ✓ | |
| | | Autoimmune thyroid disease | ✓ | ✓ | |
| | | Graft-versus-host disease | ✓ | ✓ | |
| | | Systemic lupus erythematosus | ✓ | ✓ | |
| | | Primary immunodeficiency | ✓ | | ✓ |
| | | Inflammatory bowel disease | ✓ | ✓ | |
| Human Diseases | Neurodegenerative disease | Huntington disease | ✓ | | ✓ |
| | | Spinocerebellar ataxia | | ✓ | |
| | | Prion disease | | ✓ | ✓ |
| | | Pathways of neurodegeneration - multiple diseases | | | ✓ |
| | | Parkinson disease | | | ✓ |
| | | Amyotrophic lateral sclerosis | | | ✓ |
| | | Alzheimer disease | | | ✓ |
| Human Diseases | Substance dependence | Amphetamine addiction | ✓ | ✓ | |
| | | Morphine addiction | ✓ | | |
| | | Cocaine addiction | ✓ | ✓ | |
| | | Alcoholism | ✓ | | ✓ |
| Human Diseases | Endocrine and metabolic disease | Type I diabetes mellitus | ✓ | ✓ | |
| | | Insulin resistance | ✓ | | |
| | | Alcoholic liver disease | ✓ | ✓ | |
| | | AGE-RAGE signaling pathway in diabetic complications | ✓ | ✓ | |
| | | Type II diabetes mellitus | ✓ | | |
| | | Cushing syndrome | ✓ | | |
| | | Non-alcoholic fatty liver disease | ✓ | ✓ | ✓ |
| Human Diseases | Cardiovascular disease | Lipid and atherosclerosis | ✓ | ✓ | ✓ |
| | | Viral myocarditis | ✓ | ✓ | ✓ |
| | | Hypertrophic cardiomyopathy | ✓ | ✓ | ✓ |
| | | Arrhythmogenic right ventricular cardiomyopathy | ✓ | ✓ | |
| | | Fluid shear stress and atherosclerosis | ✓ | ✓ | |
| | | Dilated cardiomyopathy | | ✓ | |

| | | | | | |
|---|---|---|---|---|---|
| | | Diabetic cardiomyopathy | | ✓ | |
| Human Diseases | Drug resistance: antineoplastic | Antifolate resistance | ✓ | | |
| | | Platinum drug resistance | ✓ | | |
| | | Endocrine resistance | ✓ | ✓ | |
| Human Diseases | Cancer: overview | Choline metabolism in cancer | ✓ | ✓ | ✓ |
| | | Viral carcinogenesis | ✓ | | |
| | | Proteoglycans in cancer | ✓ | ✓ | |
| | | Chemical carcinogenesis - reactive oxygen species | ✓ | ✓ | |
| | | MicroRNAs in cancer | ✓ | | |
| | | Chemical carcinogenesis - receptor activation | ✓ | ✓ | ✓ |
| | | Transcriptional misregulation in cancer | ✓ | | |
| | | Pathways in cancer | ✓ | ✓ | |
| | | PD-L1 expression and PD-1 checkpoint pathway in cancer | | ✓ | |
| Human Diseases | Cancer: specific types | Thyroid cancer | ✓ | | |
| | | Glioma | ✓ | | |
| | | Non-small cell lung cancer | ✓ | | |
| | | Endometrial cancer | ✓ | | |
| | | Pancreatic cancer | ✓ | ✓ | |
| | | Acute myeloid leukemia | ✓ | ✓ | |
| | | Colorectal cancer | ✓ | ✓ | |
| | | Prostate cancer | ✓ | | |
| | | Hepatocellular carcinoma | ✓ | | |
| | | Melanoma | ✓ | | |
| | | Bladder cancer | ✓ | ✓ | |
| | | Breast cancer | ✓ | | |
| | | Gastric cancer | ✓ | | |
| | | Renal cell carcinoma | ✓ | | |
| | | Chronic myeloid leukemia | ✓ | ✓ | |
| **Genetic Information Processing** | Chromosome | ATP-dependent chromatin remodeling | ✓ | ✓ | |
| Genetic Information Processing | Folding, sorting and degradation | Proteasome | ✓ | | ✓ |
| | | Ubiquitin mediated proteolysis | ✓ | ✓ | |
| | | RNA degradation | | ✓ | |
| Genetic Information Processing | Information processing in viruses | Viral life cycle - HIV-1 | ✓ | | |
| Genetic Information Processing | Replication and repair | Nucleotide excision repair | ✓ | ✓ | ✓ |
| | | Fanconi anemia pathway | ✓ | ✓ | ✓ |
| | | Base excision repair | ✓ | ✓ | |
| | | DNA replication | | ✓ | ✓ |
| | | Homologous recombination | | ✓ | ✓ |
| Genetic Information Processing | Transcription | RNA polymerase | ✓ | ✓ | ✓ |
| | | Spliceosome | ✓ | ✓ | ✓ |
| | | Basal transcription factors | | ✓ | |
| Genetic Information Processing | Translation | Ribosome biogenesis in eukaryotes | ✓ | ✓ | |
| | | Aminoacyl-tRNA biosynthesis | ✓ | ✓ | |
| | | Ribosome | ✓ | ✓ | ✓ |
| | | Nucleocytoplasmic transport | | ✓ | |

| | | | | | |
|---|---|---|---|---|---|
| | | mRNA surveillance pathway | | ✓ | |
| **Metabolism** | Amino acid metabolism | Valine, leucine and isoleucine degradation | ✓ | ✓ | |
| | | Arginine and proline metabolism | ✓ | ✓ | ✓ |
| | | Tryptophan metabolism | ✓ | ✓ | |
| | | Glycine, serine and threonine metabolism | | ✓ | |
| | | Lysine degradation | | ✓ | ✓ |
| Metabolism | Carbohydrate metabolism | Citrate cycle (TCA cycle) | ✓ | | |
| | | Pyruvate metabolism | ✓ | | |
| | | Propanoate metabolism | ✓ | | |
| | | Glycolysis / Gluconeogenesis | ✓ | | ✓ |
| | | Nucleotide metabolism | ✓ | | |
| | | Fructose and mannose metabolism | ✓ | ✓ | ✓ |
| | | Butanoate metabolism | ✓ | | |
| | | Polycomb repressive complex | ✓ | | |
| | | Starch and sucrose metabolism | | ✓ | ✓ |
| Metabolism | Energy metabolism | Oxidative phosphorylation | | | ✓ |
| Metabolism | Glycan biosynthesis and metabolism | Amino sugar and nucleotide sugar metabolism | ✓ | | ✓ |
| | | Other types of O-glycan biosynthesis | ✓ | | |
| Metabolism | Lipid metabolism | Fatty acid degradation | ✓ | | |
| | | Ether lipid | ✓ | | |
| | | Sphingolipid metabolism | | ✓ | |
| | | Glycerophospholipid metabolism | | ✓ | |
| Metabolism | Metabolism of cofactors and vitamins | Thiamine | ✓ | | |
| | | Porphyrin metabolism | | ✓ | |
| Metabolism | Metabolism of other amino acids | Glutathione metabolism | | | ✓ |
| Metabolism | Nucleotide metabolism | Purine | ✓ | | |
| **Organismal Systems** | Immune system | Cytosolic DNA-sensing pathway | ✓ | ✓ | ✓ |
| | | NOD-like receptor signaling pathway | ✓ | ✓ | ✓ |
| | | C-type lectin receptor signaling pathway | ✓ | ✓ | ✓ |
| | | Antigen processing and presentation | ✓ | ✓ | ✓ |
| | | Fc gamma R-mediated phagocytosis | ✓ | ✓ | |
| | | IL-17 signaling pathway | ✓ | ✓ | |
| | | Th1 and Th2 cell differentiation | ✓ | ✓ | |
| | | Platelet activation | ✓ | ✓ | ✓ |
| | | Leukocyte transendothelial migration | ✓ | ✓ | |
| | | RIG-I-like receptor signaling pathway | ✓ | ✓ | ✓ |
| | | T cell receptor signaling pathway | ✓ | ✓ | |
| | | Neutrophil extracellular trap formation | ✓ | ✓ | |
| | | Chemokine signaling pathway | ✓ | ✓ | |
| | | Toll-like receptor signaling pathway | ✓ | ✓ | |
| | | Fc epsilon RI signaling pathway | ✓ | ✓ | |
| | | Toll and Imd signaling pathway | ✓ | ✓ | |
| | | Intestinal immune network for IgA production | | ✓ | |
| | | Natural killer cell mediated cytotoxicity | | ✓ | |

| | | | | | |
|---|---|---|---|---|---|
| | | Hematopoietic cell lineage | | ✓ | |
| | | Th17 cell differentiation | | ✓ | |
| | | t receptor signaling pathway | | ✓ | ✓ |
| Organismal Systems | Nervous system | Cholinergic synapse | ✓ | ✓ | |
| | | Long-term potentiation | ✓ | | ✓ |
| | | Long-term depression | ✓ | | |
| | | GABAergic synapse | ✓ | ✓ | |
| | | Serotonergic synapse | ✓ | ✓ | ✓ |
| | | Neurotrophin signaling pathway | ✓ | ✓ | |
| | | EGFR tyrosine kinase inhibitor resistance | ✓ | | |
| | | Glutamatergic synapse | ✓ | | |
| | | Dopaminergic synapse | ✓ | ✓ | ✓ |
| | | Retrograde endocannabinoid signaling | ✓ | | |
| Organismal Systems | Sensory system | Inflammatory mediator regulation of TRP channels | ✓ | ✓ | |
| Organismal Systems | Aging | Longevity regulating pathway - multiple species | ✓ | | ✓ |
| | | Longevity regulating pathway | ✓ | | |
| Organismal Systems | Circulatory system | Adrenergic signaling in cardiomyocytes | ✓ | | ✓ |
| | | Vascular smooth muscle contraction | ✓ | | ✓ |
| | | Cardiac muscle contraction | | ✓ | ✓ |
| Organismal Systems | Development and regeneration | Dorso-ventral axis formation | ✓ | | |
| | | Osteoclast differentiation | ✓ | ✓ | |
| | | Axon guidance | ✓ | ✓ | |
| | | Axon regeneration | ✓ | ✓ | |
| Organismal Systems | Digestive system | Pancreatic secretion | ✓ | ✓ | |
| | | Bile secretion | ✓ | | |
| | | Salivary secretion | | | ✓ |
| | | Gastric acid secretion | | | ✓ |
| Organismal Systems | Endocrine system | Thyroid hormone signaling pathway | ✓ | | |
| | | Renin secretion | ✓ | | |
| | | Thyroid hormone synthesis | ✓ | | |
| | | Glucagon signaling pathway | ✓ | | ✓ |
| | | PPAR signaling pathway | ✓ | | |
| | | Oxytocin signaling pathway | ✓ | ✓ | ✓ |
| | | Aldosterone synthesis and secretion | ✓ | ✓ | ✓ |
| | | Insulin secretion | ✓ | | ✓ |
| | | Cortisol synthesis and secretion | ✓ | ✓ | |
| | | Ovarian steroidogenesis | ✓ | ✓ | |
| | | Adipocytokine signaling pathway | ✓ | ✓ | |
| | | Insulin signaling pathway | ✓ | ✓ | |
| | | Estrogen signaling pathway | ✓ | | |
| | | Regulation of lipolysis in adipocytes | ✓ | | |
| | | Prolactin signaling pathway | ✓ | ✓ | |
| | | Parathyroid hormone synthesis, secretion and action | ✓ | ✓ | ✓ |
| | | Progesterone-mediated oocyte maturation | ✓ | | |
| | | Melanogenesis | ✓ | ✓ | |

| | | | | | |
|---|---|---|---|---|---|
| | | GnRH signaling pathway | ✓ | ✓ | |
| | | Relaxin signaling pathway | ✓ | ✓ | |
| | | Growth hormone synthesis, secretion and action | ✓ | ✓ | |
| Organismal Systems | Environmental adaptation | Circadian entrainment | ✓ | | |
| | | Thermogenesis | ✓ | ✓ | ✓ |
| | | Circadian rhythm | ✓ | ✓ | ✓ |
| Organismal Systems | Excretory system | Endocrine and other factor-regulated calcium reabsorption | ✓ | | |
| **Cellular Processes** | Cell growth and death | Necroptosis | ✓ | ✓ | ✓ |
| | | Cell cycle | ✓ | | ✓ |
| | | Apoptosis - fly | ✓ | ✓ | |
| | | Oocyte meiosis | ✓ | | |
| | | Cell cycle - yeast | | ✓ | ✓ |
| | | Meiosis - yeast | | | ✓ |
| Cellular Processes | Cell motility | Motor proteins | ✓ | ✓ | ✓ |
| | | Regulation of actin cytoskeleton | ✓ | ✓ | |
| Cellular Processes | Cellular community - eukaryotes | Gap junction | ✓ | | |
| | | Focal adhesion | ✓ | ✓ | |
| | | Tight junction | ✓ | | ✓ |
| | | Adherens junction | ✓ | ✓ | |
| | | Signaling pathways regulating pluripotency of stem cells | ✓ | ✓ | |
| Cellular Processes | Transport and catabolism | Phagosome | ✓ | ✓ | |
| | | Peroxisome | ✓ | | |
| | | Autophagy - yeast | ✓ | ✓ | |
| | | Endocytosis | ✓ | | ✓ |
| | | Mitophagy - animal | | | ✓ |
| **Environmental Information Processing** | Membrane transport | GnRH secretion | ✓ | | |
| | | ABC transporters | | ✓ | ✓ |
| Environmental Information Processing | Signal transduction | NF-kappa B signaling pathway | ✓ | ✓ | |
| | | Hedgehog signaling pathway | ✓ | | |
| | | HIF-1 signaling pathway | ✓ | | |
| | | MAPK signaling pathway - yeast | ✓ | | |
| | | mTOR signaling pathway | ✓ | | |
| | | cGMP-PKG signaling pathway | ✓ | | |
| | | Notch signaling pathway | ✓ | | |
| | | JAK-STAT signaling pathway | ✓ | ✓ | |
| | | Calcium signaling pathway | ✓ | ✓ | ✓ |
| | | PI3K-Akt signaling pathway | ✓ | ✓ | |
| | | AMPK signaling pathway | ✓ | ✓ | ✓ |
| | | ErbB signaling pathway | ✓ | ✓ | |
| | | Hippo signaling pathway - fly | ✓ | | |
| | | Apelin signaling pathway | ✓ | ✓ | |
| | | Hippo signaling pathway - multiple species | ✓ | | |
| | | MAPK signaling pathway | ✓ | ✓ | |
| | | Hippo signaling pathway | ✓ | ✓ | |
| | | Ras signaling pathway | ✓ | ✓ | |

| | | | | | |
|---|---|---|---|---|---|
| | | cAMP signaling pathway | ✓ | ✓ | ✓ |
| | | FoxO signaling pathway | ✓ | ✓ | |
| | | Sphingolipid signaling pathway | ✓ | ✓ | ✓ |
| | | Phospholipase D signaling pathway | ✓ | ✓ | |
| | | TNF signaling pathway | ✓ | ✓ | |
| | | Rap1 signaling pathway | ✓ | ✓ | |
| | | VEGF signaling pathway | ✓ | ✓ | |
| | | MAPK signaling pathway - fly | ✓ | ✓ | |
| | | TGF-beta signaling pathway | ✓ | | |
| | | Wnt signaling pathway | ✓ | ✓ | |
| | | Phosphatidylinositol signaling system | | ✓ | ✓ |
| Environmental Information Processing | Signaling molecules and interaction | Cell adhesion molecules | ✓ | ✓ | |
| | | Viral protein interaction with cytokine and cytokine receptor | ✓ | ✓ | |
| | | Cytokine-cytokine receptor interaction | ✓ | ✓ | |
| | | Neuroactive ligand-receptor interaction | ✓ | ✓ | |

**Table S7.** Enriched pathway categories on KEGG databases for SI vs. SNI groups at 3 hpi, 2 and 14 dpi. SI: susceptible infected fish; SNI: susceptible non-infected fish; hpi: hours post infection; dpi: days post infection.

| Pathway top category | Pathway class | Enriched pathway | 3 hpi | 2 dpi | 14 dpi |
|---|---|---|---|---|---|
| **Human Diseases** | Infectious disease: viral | Epstein-Barr virus infection | ✓ | | |
| | | Influenza A | ✓ | ✓ | ✓ |
| | | Coronavirus disease - COVID-19 | ✓ | ✓ | ✓ |
| | | Herpes simplex virus 1 infection | ✓ | | |
| | | Human cytomegalovirus infection | | ✓ | ✓ |
| | | Human T-cell leukemia virus 1 infection | ✓ | | |
| | | Measles | | ✓ | |
| | | Hepatitis B | ✓ | | |
| Human Diseases | Infectious disease: bacterial | Pertussis | ✓ | | ✓ |
| | | Yersinia infection | ✓ | ✓ | ✓ |
| | | Salmonella infection | ✓ | ✓ | ✓ |
| | | Pathogenic Escherichia coli infection | ✓ | ✓ | ✓ |
| | | Shigellosis | | | ✓ |
| | | Staphylococcus aureus infection | ✓ | ✓ | ✓ |
| | | Legionellosis | | ✓ | |
| | | Bacterial invasion of epithelial cells | ✓ | | |
| | | Tuberculosis | ✓ | | |
| | | Vibrio cholerae infection | ✓ | | |
| | | Epithelial cell signaling in Helicobacter pylori infection | ✓ | | |
| Human Diseases | Infectious disease: parasitic | Amoebiasis | ✓ | | |
| | | Malaria | ✓ | | |
| | | Chagas disease | ✓ | | |
| | | Leishmaniasis | ✓ | ✓ | |
| | | Toxoplasmosis | ✓ | | |
| Human Diseases | Immune disease | Allograft rejection | ✓ | ✓ | |
| | | Autoimmune thyroid disease | ✓ | ✓ | |
| | | Graft-versus-host disease | ✓ | ✓ | |

| | | | | | |
|---|---|---|---|---|---|
| | | Systemic lupus erythematosus | ✓ | ✓ | |
| | | Primary immunodeficiency | | | ✓ |
| | | Inflammatory bowel disease | ✓ | | |
| | | Rheumatoid arthritis | ✓ | | |
| Human Diseases | Neurodegenerative disease | Huntington disease | ✓ | ✓ | ✓ |
| | | Prion disease | ✓ | | ✓ |
| | | Pathways of neurodegeneration - multiple diseases | ✓ | | ✓ |
| | | Parkinson disease | ✓ | | ✓ |
| | | Amyotrophic lateral sclerosis | ✓ | | ✓ |
| | | Alzheimer disease | ✓ | | ✓ |
| Human Diseases | Substance dependence | Amphetamine addiction | ✓ | | |
| | | Morphine addiction | ✓ | | |
| | | Cocaine addiction | ✓ | | |
| | | Alcoholism | ✓ | ✓ | ✓ |
| Human Diseases | Endocrine and metabolic disease | Type I diabetes mellitus | ✓ | | |
| | | Insulin resistance | ✓ | | |
| | | Alcoholic liver disease | | ✓ | |
| | | AGE-RAGE signaling pathway in diabetic complications | ✓ | | |
| | | Cushing syndrome | ✓ | | |
| | | Non-alcoholic fatty liver disease | | | ✓ |
| Human Diseases | Cardiovascular disease | Lipid and atherosclerosis | ✓ | | ✓ |
| | | Viral myocarditis | ✓ | ✓ | ✓ |
| | | Hypertrophic cardiomyopathy | ✓ | | ✓ |
| | | Arrhythmogenic right ventricular cardiomyopathy | ✓ | | |
| | | Fluid shear stress and atherosclerosis | ✓ | | |
| | | Dilated cardiomyopathy | ✓ | | |
| | | Diabetic cardiomyopathy | | | ✓ |
| Human Diseases | Drug resistance: antineoplastic | Endocrine resistance | ✓ | | |
| Human Diseases | Cancer: overview | Choline metabolism in cancer | ✓ | | ✓ |
| | | Chemical carcinogenesis - DNA adducts | ✓ | | |
| | | Central carbon metabolism in cancer | ✓ | | |
| | | Viral carcinogenesis | ✓ | | |
| | | Proteoglycans in cancer | ✓ | | |
| | | Chemical carcinogenesis - reactive oxygen species | | | ✓ |
| | | MicroRNAs in cancer | ✓ | | |
| | | Chemical carcinogenesis - receptor activation | ✓ | | |
| | | Transcriptional misregulation in cancer | ✓ | | |
| | | Pathways in cancer | ✓ | | |
| | | PD-L1 expression and PD-1 checkpoint pathway in cancer | ✓ | | |
| Human Diseases | Cancer: specific types | Thyroid cancer | ✓ | | |
| | | Small cell lung cancer | ✓ | | |
| | | Glioma | ✓ | | |
| | | Non-small cell lung cancer | ✓ | | |

| | | | | | |
|---|---|---|---|---|---|
| | | Endometrial cancer | ✓ | | |
| | | Pancreatic cancer | ✓ | | |
| | | Acute myeloid leukemia | ✓ | | |
| | | Colorectal cancer | ✓ | | |
| | | Prostate cancer | ✓ | | |
| | | Hepatocellular carcinoma | ✓ | | |
| | | Bladder cancer | ✓ | | |
| | | Breast cancer | ✓ | | |
| | | Gastric cancer | ✓ | | |
| | | Renal cell carcinoma | ✓ | | |
| | | Chronic myeloid leukemia | ✓ | | |
| **Genetic Information Processing** | Chromosome | ATP-dependent chromatin remodeling | ✓ | | |
| Genetic Information Processing | Folding, sorting and degradation | Proteasome | ✓ | | ✓ |
| | | Ubiquitin mediated proteolysis | ✓ | | |
| | | Protein processing in endoplasmic reticulum | | ✓ | |
| Genetic Information Processing | Replication and repair | Nucleotide excision repair | ✓ | | ✓ |
| | | Fanconi anemia pathway | ✓ | | ✓ |
| | | Base excision repair | ✓ | | |
| | | DNA replication | | | ✓ |
| | | Homologous recombination | | | ✓ |
| Genetic Information Processing | Transcription | RNA polymerase | ✓ | | ✓ |
| | | Spliceosome | ✓ | | ✓ |
| | | Basal transcription factors | ✓ | | |
| | | Aminoacyl-tRNA biosynthesis | ✓ | | |
| | | Ribosome | ✓ | ✓ | ✓ |
| | | Nucleocytoplasmic transport | ✓ | | |
| **Metabolism** | Amino acid metabolism | Alanine, aspartate and glutamate metabolism | | ✓ | |
| | | Arginine and proline metabolism | ✓ | | ✓ |
| | | Lysine degradation | ✓ | | ✓ |
| Metabolism | Carbohydrate metabolism | Inositol phosphate metabolism | ✓ | | |
| | | Pyruvate metabolism | ✓ | ✓ | |
| | | Glycolysis / Gluconeogenesis | | | ✓ |
| | | Fructose and mannose metabolism | | | ✓ |
| | | Polycomb repressive complex | ✓ | | |
| | | Starch and sucrose metabolism | ✓ | | ✓ |
| Metabolism | Energy metabolism | Oxidative phosphorylation | ✓ | | ✓ |
| | | Carbon fixation in photosynthetic organisms | ✓ | | |
| Metabolism | Glycan biosynthesis and metabolism | Amino sugar and nucleotide sugar metabolism | | ✓ | ✓ |
| Metabolism | Lipid metabolism | Steroid hormone biosynthesis | ✓ | ✓ | |
| | | Sphingolipid metabolism | ✓ | | |
| | | Glycerophospholipid metabolism | | ✓ | |
| Metabolism | Metabolism of cofactors and vitamins | Thiamine metabolism | | ✓ | |
| | | Porphyrin metabolism | ✓ | | |
| | | Retinol metabolism | ✓ | | |

| | | | | | |
|---|---|---|---|---|---|
| Metabolism | Metabolism of other amino acids | Glutathione metabolism | ✓ | | ✓ |
| Metabolism | Nucleotide metabolism | Purine | ✓ | | |
| | | Citrate cycle (TCA cycle) | ✓ | | |
| Metabolism | Xenobiotics biodegradation and metabolism | Metabolism of xenobiotics by cytochrome P450 | ✓ | | |
| | | Drug metabolism - other enzymes | ✓ | | |
| | | Drug metabolism - cytochrome P450 | ✓ | | |
| **Organismal Systems** | Immune system | Cytosolic DNA-sensing pathway | ✓ | ✓ | ✓ |
| | | Complement and coagulation cascades | ✓ | ✓ | |
| | | NOD-like receptor signaling pathway | ✓ | ✓ | ✓ |
| | | C-type lectin receptor signaling pathway | ✓ | ✓ | ✓ |
| | | Antigen processing and presentation | ✓ | | ✓ |
| | | Fc gamma R-mediated phagocytosis | ✓ | | |
| | | IL-17 signaling pathway | ✓ | ✓ | |
| | | Th1 and Th2 cell differentiation | ✓ | | |
| | | Platelet activation | ✓ | | ✓ |
| | | Leukocyte transendothelial migration | ✓ | | |
| | | RIG-I-like receptor signaling pathway | | | ✓ |
| | | T cell receptor signaling pathway | ✓ | | |
| | | Neutrophil extracellular trap formation | ✓ | ✓ | |
| | | Chemokine signaling pathway | ✓ | | |
| | | Toll-like receptor signaling pathway | ✓ | | |
| | | Fc epsilon RI signaling pathway | ✓ | | |
| | | Toll and Imd signaling pathway | ✓ | | |
| | | Th17 cell differentiation | ✓ | | |
| | | B cell receptor signaling pathway | ✓ | | ✓ |
| Organismal Systems | Nervous system | Cholinergic synapse | ✓ | | |
| | | Long-term potentiation | ✓ | | ✓ |
| | | Long-term depression | ✓ | ✓ | |
| | | GABAergic synapse | ✓ | | |
| | | Serotonergic synapse | ✓ | | ✓ |
| | | Neurotrophin signaling pathway | ✓ | | |
| | | EGFR tyrosine kinase inhibitor resistance | ✓ | | |
| | | Glutamatergic synapse | ✓ | | |
| | | Dopaminergic synapse | ✓ | | ✓ |
| Organismal Systems | Sensory system | Inflammatory mediator regulation of TRP channels | ✓ | | |
| Organismal Systems | Aging | Longevity regulating pathway | ✓ | | ✓ |
| Organismal Systems | Circulatory system | Adrenergic signaling in cardiomyocytes | ✓ | | ✓ |
| | | Vascular smooth muscle contraction | ✓ | | ✓ |
| | | Cardiac muscle contraction | ✓ | | ✓ |
| Organismal Systems | Development and regeneration | Dorso-ventral axis formation | ✓ | | |
| | | Osteoclast differentiation | ✓ | | |
| | | Axon guidance | ✓ | | |
| | | Axon regeneration | ✓ | | |
| | Digestive system | Vitamin digestion and absorption | | ✓ | |

| | | | | | |
|---|---|---|---|---|---|
| Organismal Systems | | Cholesterol metabolism | | ✓ | |
| | | Salivary secretion | | | ✓ |
| | | Gastric acid secretion | ✓ | | ✓ |
| Organismal Systems | Endocrine system | Thyroid hormone signaling pathway | ✓ | | |
| | | Renin-angiotensin system | ✓ | | |
| | | Glucagon signaling pathway | ✓ | | ✓ |
| | | Oxytocin signaling pathway | ✓ | | ✓ |
| | | Aldosterone synthesis and secretion | ✓ | | ✓ |
| | | Insulin secretion | | | ✓ |
| | | Cortisol synthesis and secretion | ✓ | | |
| | | Adipocytokine signaling pathway | ✓ | | |
| | | Insulin signaling pathway | ✓ | | |
| | | Estrogen signaling pathway | ✓ | ✓ | |
| | | Prolactin signaling pathway | ✓ | | |
| | | Parathyroid hormone synthesis, secretion and action | ✓ | | ✓ |
| | | Progesterone-mediated oocyte maturation | ✓ | | |
| | | Melanogenesis | ✓ | | |
| | | GnRH signaling pathway | ✓ | | |
| | | Relaxin signaling pathway | ✓ | | |
| | | Growth hormone synthesis, secretion and action | ✓ | | |
| Organismal Systems | Environmental adaptation | Circadian entrainment | ✓ | | |
| | | Thermogenesis | ✓ | | ✓ |
| | | Circadian rhythm | ✓ | | ✓ |
| **Cellular Processes** | Cell growth and death | Necroptosis | ✓ | ✓ | ✓ |
| | | p53 signaling pathway | ✓ | | |
| | | Cell cycle | ✓ | | ✓ |
| | | Apoptosis - fly | ✓ | | |
| | | Oocyte meiosis | ✓ | | |
| | | Cell cycle - yeast | | | ✓ |
| | | Meiosis - yeast | | | ✓ |
| Cellular Processes | Cell motility | Motor proteins | ✓ | | ✓ |
| | | Regulation of actin cytoskeleton | ✓ | ✓ | |
| Cellular Processes | Cellular community - eukaryotes | Gap junction | ✓ | ✓ | |
| | | Focal adhesion | ✓ | | |
| | | Tight junction | ✓ | | ✓ |
| | | Adherens junction | ✓ | | |
| | | Signaling pathways regulating pluripotency of stem cells | ✓ | | |
| Cellular Processes | Transport and catabolism | Phagosome | ✓ | | |
| | | Peroxisome | ✓ | | |
| | | Autophagy - yeast | ✓ | | |
| | | Lysosome | ✓ | ✓ | |
| | | Endocytosis | | | ✓ |
| | | Autophagy - animal | ✓ | | |
| | | Mitophagy - animal | ✓ | | ✓ |
| **Environmental Information Processing** | Membrane transport | GnRH secretion | ✓ | | |
| | | ABC transporters | | ✓ | ✓ |

| | | | | | |
|---|---|---|---|---|---|
| Environmental Information Processing | Signal transduction | MAPK signaling pathway - yeast | ✓ | | |
| | | cGMP-PKG signaling pathway | ✓ | | |
| | | Notch signaling pathway | ✓ | | |
| | | JAK-STAT signaling pathway | ✓ | | |
| | | Calcium signaling pathway | | | ✓ |
| | | PI3K-Akt signaling pathway | ✓ | | |
| | | AMPK signaling pathway | ✓ | | ✓ |
| | | ErbB signaling pathway | ✓ | | |
| | | Hippo signaling pathway - fly | ✓ | | |
| | | Apelin signaling pathway | ✓ | | |
| | | Hippo signaling pathway - multiple species | ✓ | | |
| | | MAPK signaling pathway | ✓ | | |
| | | Hippo signaling pathway | ✓ | | |
| | | Ras signaling pathway | ✓ | | |
| | | cAMP signaling pathway | ✓ | | ✓ |
| | | FoxO signaling pathway | ✓ | | |
| | | Sphingolipid signaling pathway | ✓ | | ✓ |
| | | Phospholipase D signaling pathway | ✓ | | |
| | | TNF signaling pathway | ✓ | | |
| | | Rap1 signaling pathway | ✓ | | |
| | | VEGF signaling pathway | ✓ | | |
| | | MAPK signaling pathway - fly | ✓ | | |
| | | TGF-beta signaling pathway | ✓ | | |
| | | Wnt signaling pathway | | ✓ | |
| | | Phosphatidylinositol signaling system | ✓ | ✓ | ✓ |
| Environmental Information Processing | Signaling molecules and interaction | Cell adhesion molecules | ✓ | ✓ | |
| | | ECM-receptor interaction | ✓ | | |

**Table S8.** Primers and probes used in the present work.

| Gene | Primer sequence (5′ - 3′) | Accession number | Product size (bp) | Reference |
|---|---|---|---|---|
| *NNV load quantification* | | | | |
| **Primers** | oPVP154: TCCAAGCCGGTCCTAGTCAA | N/A | 168/171 | [103] |
| | oPVP155: CACGAACGTKCGCATCTCGT | | | |
| **Taqman probe** | tqPVP16: Cy5-CGATCGATCAGCACCTSGTCBHQ2 | N/A | N/A | [103] |
| *qPCR validation* | | | | |
| **aldoaa** | Up: | | | Present work |
| | Dp: | | | |
| **b4galt1** | Up: CAGCTCGTGGGTCCTCTCTAT | XM_051420093.1 | 142 | Present work |
| | Dp: CAGCTCGTGGGTCCTCTCTAT | | | |
| **cldn5b** | Up: | | | Present work |
| | Dp: | | | |
| **iglc** | Up: CGTCAGACTGGAACATGGATAA | XM_051395256.1 | 121 | Present work |
| | Dp: GTCATTCTGCTCCTCTGCTATT | | | |
| **nlrc3l** | Up: GATGACAGCAGTGGACCTTT | XM_051394170.1 | 91 | Present work |
| | Dp: GATGTCGGGCTTCTTCAGATAC | | | |
| **nlrp12** | Up: | | | Present work |
| | Dp: | | | |

| | | | | |
|---|---|---|---|---|
| **ppm1k** | Up: GGAGGAAGATGATCTGGAGAAAG<br>Dp: AGGAGGCGTTGTTGAAGTAG | XM_051393798.1 | 95 | Present work |
| **tnni2a.1** | Up: GCTCCAAACACACGGTCAAC<br>Dp: AGTCACCAATGTCACGCAGT | XM_051381102.1 | 99 | Present work |
| **ttnh** | Up: ACTCCACCAACAACTCCATTAG<br>Dp: CCATTCTGTCTCTTCTCCCTTTAC | XM_051386701.1 | 110 | Present work |
| **zmym1** | Up: ACCGAACTGTCCCTCATCTA<br>Dp: AGTGCTCTGAAGGTTGTTCTC | XM_051377851.1 | 99 | Present work |
| *Reference* | | | | |
| **b-actin** | Up: GATCTGGCATCACACCTTCTAC<br>Dp: TCTTCTCCCTGTTGGCTTTG | AJ537421.1 | 104 | [36] |

N/A: Non applicable.

## References

36. Toubanaki, D.K.; Efstathiou, A.; Tzortzatos, O.P.; Valsamidis, M.A.; Papaharisis, L.; Bakopoulos, V.; Karagouni, E. Nervous Necrosis Virus Modulation of European Sea Bass (*Dicentrarchus labrax*, L.) Immune Genes and Transcriptome towards Establishment of Virus Carrier State. *Int. J. Mol. Sci.* **2023**, *24*, 16613. https://doi.org/10.3390/ijms242316613.
103. Baud, M.; Cabon, J.; Salomoni, A.; Toffan, A.; Panzarin, V.; Bigarré, L. First generic one step real-time Taqman RT-PCR targeting the RNA1 of betanodaviruses. *J. Virol. Methods* **2015**, *211*, 1–7. https://doi.org/10.1016/j.jviromet.2014.09.016.

**Disclaimer/Publisher's Note:** The statements, opinions and data contained in all publications are solely those of the individual author(s) and contributor(s) and not of MDPI and/or the editor(s). MDPI and/or the editor(s) disclaim responsibility for any injury to people or property resulting from any ideas, methods, instructions or products referred to in the content.
